# Supplementary material for: Mendelian randomization analysis using mixture models for robust and efficient estimation of causal effects
Source: Nat Commun. 2019 Apr 26;10:1941. doi: 10.1038/s41467-019-09432-2 (PMC6486646; doi:10.1038/s41467-019-09432-2)
Supplement: Supplementary file 1 — Supplementary Information [file 41467_2019_9432_MOESM1_ESM.pdf]

Mendelian Randomization Analysis Using Mixture Models for Robust and Efficient Estimation of Causal Effects

Qi and Chatterjee

Supplementary Notes

1 MRMix estimator

In this section, we give the details on the derivation of MRMix estimator. We first introduce a set of notations. Denote by $\hat{\beta}_{jx}$ and $\hat{\beta}_{jy}$ the GWAS estimates of the effects of SNP j on exposure X and outcome Y, respectively. Let s_{jx} and s_{jy} be the corresponding standard errors, which are considered as known constants. The causal effect of X on Y, denoted by θ, is our quantity of interest. Denote by u_{jx} and u_{jy} the direct effect of SNP j on X and Y, i.e. the effects not mediated by the other variable. The GWAS effect size β_{jx} and β_{jy} satisfies

$$\beta_{jx} = u_{jx}, \quad \beta_{jy} = u_{jy} + \theta u_{jx}.$$

In the rest of this section, we drop subscript j for simple notations.

We model the joint distribution of u_x and u_y using a bivariate mixture model with the following components (**Figure 1a**):

1. $u_x \sim N(0, \sigma_x^2)$, $u_y = 0$ (valid IVs).
2. $\binom{u_x}{u_y} \sim N\left(\binom{0}{0}, \begin{pmatrix} \sigma_x^2 & \sigma_{xy} \\ \sigma_{xy} & \sigma_y^2 \end{pmatrix}\right)$ (horizontal pleiotropy).
3. $u_x = 0$, $u_y \sim N(0, \sigma_y^2)$.
4. $u_x = u_y = 0$.

Denote the mixture probabilities of case 1-4 by $\pi_1, \pi_2, \pi_3, \pi_4$ respectively. The model can also be written as

$$\begin{pmatrix} u_x \\ u_y \end{pmatrix} \sim \pi_1 \begin{pmatrix} N(0, \sigma_x^2) \\ \delta_0 \end{pmatrix} + \pi_2 N\left(\begin{pmatrix} 0 \\ 0 \end{pmatrix}, \begin{pmatrix} \sigma_x^2 & \sigma_{xy} \\ \sigma_{xy} & \sigma_y^2 \end{pmatrix}\right) + \pi_3 \begin{pmatrix} \delta_0 \\ N(0, \sigma_y^2) \end{pmatrix} + \pi_4 \begin{pmatrix} \delta_0 \\ \delta_0 \end{pmatrix},$$

where δ_0 is the point mass at 0.

With θ being the true causal effect, the residual $\beta_y - \theta\beta_x$ satisfies

$$\beta_y - \theta\beta_x = (u_y + \theta u_x) - \theta u_x = u_y,$$

which have the following distribution under cases 1-4:

1. $\beta_y - \theta\beta_x = 0$.

2. $\beta_y - \theta\beta_x = u_y \sim N(0, \sigma_y^2)$.

3. $\beta_y - \theta\beta_x = u_y \sim N(0, \sigma_y^2)$.

4. $\beta_y - \theta\beta_x = 0$.

This holds because u_y is exactly 0 in scenarios 1 and 4, and has distribution $N(0, \sigma_y^2)$ in scenarios 2 and 3. Therefore, $\beta_y - \theta\beta_x \sim (\pi_1 + \pi_4)\delta_0 + (\pi_2 + \pi_3)N(0, \sigma_y^2)$ if θ is the true value. Under a different value $\tilde{\theta}$, The working residual $\beta_y - \tilde{\theta}\beta_x$ also has a mixture normal distribution, with less point mass at zero:

$$\begin{aligned}\beta_y - \tilde{\theta}\beta_x =& (\beta_y - \theta\beta_x) + (\theta - \tilde{\theta})\beta_x = u_y + (\theta - \tilde{\theta})u_x \\ =& \pi_1 N(0, (\theta - \tilde{\theta})^2\sigma_x^2) + \pi_2 N(0, \sigma_y^2 + 2(\theta - \tilde{\theta})\sigma_{xy} + (\theta - \tilde{\theta})^2\sigma_x^2) + \pi_3 N(0, \sigma_y^2) + \pi_4\delta_0\end{aligned}$$

In GWAS, we obtain noised estimates $\hat{\beta}_x$ and $\hat{\beta}_y$ of the effect sizes β_x and β_y. Assume that the GWAS for X and Y have no overlapping subjects (two-sample MR). Then the corresponding distributions are

$$\begin{aligned}\hat{\beta}_y - \theta\hat{\beta}_x \sim& (\pi_1 + \pi_4)N\Big(0, s_y^2 + \theta^2 s_x^2\Big) + (\pi_2 + \pi_3)N\Big(0, \sigma_y^2 + s_y^2 + \theta^2 s_x^2\Big) \\ \hat{\beta}_y - \tilde{\theta}\hat{\beta}_x \sim& \pi_1 N\Big(0, (\theta - \tilde{\theta})^2\sigma_x^2 + s_y^2 + \tilde{\theta}^2 s_x^2\Big) + \pi_2 N\Big(0, \sigma_y^2 + 2(\theta - \tilde{\theta})\sigma_{xy} + (\theta - \tilde{\theta})^2\sigma_x^2 + s_y^2 + \tilde{\theta}^2 s_x^2\Big) \\ &+ \pi_3 N\Big(0, \sigma_y^2 + s_y^2 + \tilde{\theta}^2 s_x^2\Big) + \pi_4 N\Big(0, s_y^2 + \tilde{\theta}^2 s_x^2\Big).\end{aligned}$$

Note that for the true causal effect θ, there is enriched probability mass $\pi_1 + \pi_4$ at the "null" component $N\Big(0, s_y^2 + \tilde{\theta}^2 s_x^2\Big)$; while for another value $\tilde{\theta}$ which is not the true causal effect, this probability mass is only π_4. This enrichment is contributed by the valid IVs. Therefore, we propose a two-step algorithm to estimate the causal effect:

1. For each fixed $\tilde{\theta}$, fit a 2-component normal mixture model

$$\hat{\beta}_y - \tilde{\theta}\hat{\beta}_x \sim \pi_0 N\Big(0, s_y^2 + \tilde{\theta}^2 s_x^2\Big) + (1 - \pi_0)N(0, \sigma^2).$$

   using EM algorithm to get the estimates of the unknown parameters as $\hat{\pi}_0(\tilde{\theta})$ and $\hat{\sigma}^2(\tilde{\theta})$.

2. Search over a grid of $\tilde{\theta}$ values and choose the one that maximizes $\hat{\pi}_0(\tilde{\theta})$ as the estimate, i.e.

$$\hat{\theta} = argmax_{\tilde{\theta}} \hat{\pi}_0(\tilde{\theta}).$$

2 Asymptotic theory for MRMix estimator

2.1 Estimating equation and variance estimator

In this section, we formulate MRMix into an estimating equation and use asymptotic theory to derive the variance of MRMix estimator. Recall that for each possible value of θ, we fit a two-component mixture normal model on the residuals

$$\hat{\beta}_{jy} - \theta\hat{\beta}_{jx} \sim \pi_0 N(0, s_{jy}^2 + \theta^2 s_{jx}^2) + (1-\pi_0)N(0, \sigma^2).$$

across SNPs $j = 1, 2, ..., M$. Here π_0 is the probability mass of the "null" component and σ^2 is the variance of the "non-null" component. Define density functions:

$$f_1(\beta_x, \beta_y, s_x, s_y; \theta) = \frac{1}{\sqrt{2\pi(s_y^2 + \theta^2 s_x^2)}} \exp\big(-\frac{(\beta_y - \theta\beta_x)^2}{2(s_y^2 + \theta^2 s_x^2)}\big) \quad (1)$$

$$f_2(\beta_x, \beta_y, s_x, s_y; \theta, \sigma^2) = \frac{1}{\sqrt{2\pi\sigma^2}} \exp\big(-\frac{(\beta_y - \theta\beta_x)^2}{2\sigma^2}\big) \quad (2)$$

$$f(\beta_x, \beta_y, s_x, s_y; \theta, \pi_0, \sigma^2) = \pi_0 f_1(\beta_x, \beta_y, s_x, s_y; \theta) + (1-\pi_0) f_2(\beta_x, \beta_y, s_x, s_y; \theta, \pi_0, \sigma^2). \quad (3)$$

Also define $\boldsymbol{\beta}_x = (\beta_{1x}, ..., \beta_{Mx})$, $\boldsymbol{\beta}_y = (\beta_{1y}, ..., \beta_{My})$, $\hat{\boldsymbol{\beta}}_x = (\hat{\beta}_{1x}, ..., \hat{\beta}_{Mx})$, $\hat{\boldsymbol{\beta}}_y = (\hat{\beta}_{1y}, ..., \hat{\beta}_{My})$, $\boldsymbol{s}_x = (s_{1x}, ..., s_{Mx})$ and $\boldsymbol{s}_y = (s_{1y}, ..., s_{My})$. Hence the log-likelihood can be written as

$$l(\theta, \pi_0, \sigma^2 | \hat{\boldsymbol{\beta}}_x, \hat{\boldsymbol{\beta}}_y, \boldsymbol{s}_x, \boldsymbol{s}_y) = \sum_j \log f(\hat{\beta}_{jx}, \hat{\beta}_{jy}, s_{jx}, s_{jy}; \theta, \pi_0, \sigma^2).$$

The score functions are

$$\frac{\partial l}{\partial \pi_0} = \sum_j \frac{f_1(\hat{\beta}_{jx}, \hat{\beta}_{jy}, s_{jx}, s_{jy}; \theta) - f_2(\hat{\beta}_{jx}, \hat{\beta}_{jy}, s_{jx}, s_{jy}; \theta, \sigma^2)}{f(\hat{\beta}_{jx}, \hat{\beta}_{jy}, s_{jx}, s_{jy}; \theta, \pi_0, \sigma^2)} \tag{4}$$

$$\frac{\partial l}{\partial \sigma^2} = (1 - \pi_0) \sum_j \frac{\frac{\partial}{\partial \sigma^2} f_2(\hat{\beta}_{jx}, \hat{\beta}_{jy}, s_{jx}, s_{jy}; \theta, \sigma^2)}{f(\hat{\beta}_{jx}, \hat{\beta}_{jy}, s_{jx}, s_{jy}; \theta, \pi_0, \sigma^2)}. \tag{5}$$

For a fixed θ, we estimate π_0 and σ^2 by solving the score equations. Therefore, $\hat{\pi}_0$ and $\hat{\sigma}^2$ are implicit functions of θ. We write them as $\hat{\pi}_0(\theta, \hat{\boldsymbol{\beta}}_x, \hat{\boldsymbol{\beta}}_y)$ and $\hat{\sigma}^2(\theta, \hat{\boldsymbol{\beta}}_x, \hat{\boldsymbol{\beta}}_y)$ to reflect that they also depend on the effect estimates $\hat{\boldsymbol{\beta}}_x$ and $\hat{\boldsymbol{\beta}}_y$. We omit $\boldsymbol{s}_x$ and $\boldsymbol{s}_y$ since they are viewed as known constants. Partial derivatives $\frac{\partial \hat{\pi}_0}{\partial \theta}$ and $\frac{\partial \hat{\sigma}^2}{\partial \theta}$ can be computed using implicit function theorem. **MRMix estimator is obtained by maximizing $\hat{\pi}_0$ over θ, which is equivalent to solving equation**

$$\frac{\partial \hat{\pi}_0}{\partial \theta} = 0.$$

We further take the partial derivative of (4) and (5) over θ:

$$0 = \frac{\partial}{\partial \theta}\Big(\frac{\partial l}{\partial \pi_0}\Big) = \frac{\partial^2 l}{\partial \pi_0 \partial \theta} + \frac{\partial^2 l}{\partial \pi_0^2}\frac{\partial \hat{\pi}_0}{\partial \theta} + \frac{\partial^2 l}{\partial \pi_0 \partial \sigma^2}\frac{\partial \hat{\sigma}^2}{\partial \theta}$$

$$0 = \frac{\partial}{\partial \theta}\Big(\frac{\partial l}{\partial \sigma^2}\Big) = \frac{\partial^2 l}{\partial \sigma^2 \partial \theta} + \frac{\partial^2 l}{\partial \sigma^2 \partial \pi_0}\frac{\partial \hat{\pi}_0}{\partial \theta} + \frac{\partial^2 l}{\partial (\sigma^2)^2}\frac{\partial \hat{\sigma}^2}{\partial \theta}.$$

Therefore

$$\begin{pmatrix} \frac{\partial \hat{\pi}_0}{\partial \theta} \\ \frac{\partial \hat{\sigma}^2}{\partial \theta} \end{pmatrix} = - \begin{pmatrix} \frac{\partial^2 l}{\partial \pi_0^2} & \frac{\partial^2 l}{\partial \pi_0 \partial \sigma^2} \\ \frac{\partial^2 l}{\partial \sigma^2 \partial \pi_0} & \frac{\partial^2 l}{\partial (\sigma^2)^2} \end{pmatrix}^{-1} \begin{pmatrix} \frac{\partial^2 l}{\partial \pi_0 \partial \theta} \\ \frac{\partial^2 l}{\partial \sigma^2 \partial \theta} \end{pmatrix}. \tag{6}$$

Solving equation $\frac{\partial \hat{\pi}_0}{\partial \theta} = 0$ is equivalent to solving

$$\frac{\partial^2 l}{\partial (\sigma^2)^2}\frac{\partial^2 l}{\partial \pi_0 \partial \theta} - \frac{\partial^2 l}{\partial \sigma^2 \partial \pi_0}\frac{\partial^2 l}{\partial \sigma^2 \partial \theta}\Big|_{\pi_0 = \hat{\pi}_0(\theta, \hat{\boldsymbol{\beta}}_x, \hat{\boldsymbol{\beta}}_y), \sigma^2 = \hat{\sigma}^2(\theta, \hat{\boldsymbol{\beta}}_x, \hat{\boldsymbol{\beta}}_y), \boldsymbol{\beta}_x = \hat{\boldsymbol{\beta}}_x, \boldsymbol{\beta}_y = \hat{\boldsymbol{\beta}}_y} = 0. \tag{7}$$

with respect to θ. **This is the estimating equation we use to derive the asymptotic formula.**

From here, define function $S(\theta, \pi_0, \sigma^2, \boldsymbol{\beta}_x, \boldsymbol{\beta}_y) = \frac{\partial^2 l}{\partial (\sigma^2)^2}\frac{\partial^2 l}{\partial \pi_0 \partial \theta} - \frac{\partial^2 l}{\partial \sigma^2 \partial \pi_0}\frac{\partial^2 l}{\partial \sigma^2 \partial \theta}$, hence $S\big(\theta, \hat{\pi}_0(\theta, \hat{\boldsymbol{\beta}}_x, \hat{\boldsymbol{\beta}}_y), \hat{\sigma}^2(\theta, \hat{\boldsymbol{\beta}}_x, \hat{\boldsymbol{\beta}}_y), \hat{\boldsymbol{\beta}}_x, \hat{\boldsymbol{\beta}}_y\big)$ is equal to the left hand side of

Equation (7). Expanding (7) w.r.t. θ, we have

$$\begin{aligned}
0 &= S\big(\hat{\theta}, \hat{\pi}_0(\hat{\theta}, \hat{\boldsymbol{\beta}}_x, \hat{\boldsymbol{\beta}}_y), \hat{\sigma}^2(\hat{\theta}, \hat{\boldsymbol{\beta}}_x, \hat{\boldsymbol{\beta}}_y), \hat{\boldsymbol{\beta}}_x, \hat{\boldsymbol{\beta}}_y\big) \\
&\approx S(\theta, \hat{\pi}_0(\theta, \hat{\boldsymbol{\beta}}_x, \hat{\boldsymbol{\beta}}_y), \hat{\sigma}^2(\theta, \hat{\boldsymbol{\beta}}_x, \hat{\boldsymbol{\beta}}_y), \hat{\boldsymbol{\beta}}_x, \hat{\boldsymbol{\beta}}_y) + \Big(\frac{\partial S}{\partial \theta} + \frac{\partial S}{\partial \pi_0}\frac{\partial \hat{\pi}_0}{\partial \theta} + \frac{\partial S}{\partial \sigma^2}\frac{\partial \hat{\sigma}^2}{\partial \theta}\Big)(\hat{\theta} - \theta)
\end{aligned}$$

Rearrange the terms and expand $S(\theta, \hat{\pi}_0(\theta, \hat{\boldsymbol{\beta}}_x, \hat{\boldsymbol{\beta}}_y), \hat{\sigma}^2(\theta, \hat{\boldsymbol{\beta}}_x, \hat{\boldsymbol{\beta}}_y), \hat{\boldsymbol{\beta}}_x, \hat{\boldsymbol{\beta}}_y)$ w.r.t. $\hat{\boldsymbol{\beta}}_x$ and $\hat{\boldsymbol{\beta}}_y$

$$\begin{aligned}
\hat{\theta} - \theta &\approx -\Big(\frac{\partial S}{\partial \theta} + \frac{\partial S}{\partial \pi_0}\frac{\partial \hat{\pi}_0}{\partial \theta} + \frac{\partial S}{\partial \sigma^2}\frac{\partial \hat{\sigma}^2}{\partial \theta}\Big)^{-1} S(\theta, \hat{\pi}_0(\theta, \hat{\boldsymbol{\beta}}_x, \hat{\boldsymbol{\beta}}_y), \hat{\sigma}^2(\theta, \hat{\boldsymbol{\beta}}_x, \hat{\boldsymbol{\beta}}_y), \hat{\boldsymbol{\beta}}_x, \hat{\boldsymbol{\beta}}_y) \\
&= -\Big(\frac{\partial S}{\partial \theta} + \frac{\partial S}{\partial \pi_0}\frac{\partial \hat{\pi}_0}{\partial \theta} + \frac{\partial S}{\partial \sigma^2}\frac{\partial \hat{\sigma}^2}{\partial \theta}\Big)^{-1} \Big\{ S(\theta, \hat{\pi}_0(\theta, \boldsymbol{\beta}_x, \boldsymbol{\beta}_y), \hat{\sigma}^2(\theta, \boldsymbol{\beta}_x, \boldsymbol{\beta}_y), \boldsymbol{\beta}_x, \boldsymbol{\beta}_y) \\
&+ \sum_{j=1}^{M} \Big(\frac{\partial S}{\partial \beta_{jx}} + \frac{\partial S}{\partial \pi_0}\frac{\partial \hat{\pi}_0}{\partial \beta_{jx}} + \frac{\partial S}{\partial \sigma^2}\frac{\partial \hat{\sigma}^2}{\partial \beta_{jx}}\Big)(\hat{\beta}_{jx} - \beta_{jx}) + \sum_{j=1}^{M} \Big(\frac{\partial S}{\partial \beta_{jy}} + \frac{\partial S}{\partial \pi_0}\frac{\partial \hat{\pi}_0}{\partial \beta_{jy}} + \frac{\partial S}{\partial \sigma^2}\frac{\partial \hat{\sigma}^2}{\partial \beta_{jy}}\Big)(\hat{\beta}_{jy} - \beta_{jy}) \Big\}
\end{aligned}$$

Therefore the asymptotic variance of $\hat{\theta}$ can be expressed as

$$\begin{aligned}
var(\hat{\theta}) &\approx \Big(\frac{\partial S}{\partial \theta} + \frac{\partial S}{\partial \pi_0}\frac{\partial \hat{\pi}_0}{\partial \theta} + \frac{\partial S}{\partial \sigma^2}\frac{\partial \hat{\sigma}^2}{\partial \theta}\Big)^{-2} \\
&\Big\{ \sum_{j=1}^{M} \Big(\frac{\partial S}{\partial \beta_{jx}} + \frac{\partial S}{\partial \pi_0}\frac{\partial \hat{\pi}_0}{\partial \beta_{jx}} + \frac{\partial S}{\partial \sigma^2}\frac{\partial \hat{\sigma}^2}{\partial \beta_{jx}}\Big)^2 s_{jx}^2 + \sum_{j=1}^{M} \Big(\frac{\partial S}{\partial \beta_{jy}} + \frac{\partial S}{\partial \pi_0}\frac{\partial \hat{\pi}_0}{\partial \beta_{jy}} + \frac{\partial S}{\partial \sigma^2}\frac{\partial \hat{\sigma}^2}{\partial \beta_{jy}}\Big)^2 s_{jy}^2 \Big\}
\end{aligned}$$

Plug in estimates $\theta = \hat{\theta}$, $\pi_0 = \hat{\pi}_0(\hat{\theta}, \hat{\boldsymbol{\beta}}_x, \hat{\boldsymbol{\beta}}_y)$ $\sigma^2 = \hat{\sigma}^2(\hat{\theta}, \hat{\boldsymbol{\beta}}_x, \hat{\boldsymbol{\beta}}_y)$, $\boldsymbol{\beta}_x = \hat{\boldsymbol{\beta}}_x$ and $\boldsymbol{\beta}_y = \hat{\boldsymbol{\beta}}_y$ to get the variance estimate.

2.2 Partial derivative functions

The partial derivatives of the function $S(\theta, \pi_0, \sigma^2, \boldsymbol{\beta}_x, \boldsymbol{\beta}_y)$ can be derived as follows. Since the derivatives for β_{jx} and β_{jy} are in the same form, we write them in terms of a unified notation $\beta_{j\cdot}$.

$$\begin{aligned}
\frac{\partial S}{\partial \theta} &= \frac{\partial^3 l}{\partial (\sigma^2)^2 \partial \theta}\frac{\partial^2 l}{\partial \pi_0 \partial \theta} + \frac{\partial^2 l}{\partial (\sigma^2)^2}\frac{\partial^3 l}{\partial \pi_0 \partial \theta^2} - \frac{\partial^3 l}{\partial \sigma^2 \partial \pi_0 \partial \theta}\frac{\partial^2 l}{\partial \sigma^2 \partial \theta} - \frac{\partial^2 l}{\partial \sigma^2 \partial \pi_0}\frac{\partial^3 l}{\partial \sigma^2 \partial \theta^2} \\
\frac{\partial S}{\partial \pi_0} &= \frac{\partial^3 l}{\partial (\sigma^2)^2 \partial \pi_0}\frac{\partial^2 l}{\partial \pi_0 \partial \theta} + \frac{\partial^2 l}{\partial (\sigma^2)^2}\frac{\partial^3 l}{\partial \pi_0^2 \partial \theta} - \frac{\partial^3 l}{\partial \sigma^2 \partial \pi_0^2}\frac{\partial^2 l}{\partial \sigma^2 \partial \theta} - \frac{\partial^2 l}{\partial \sigma^2 \partial \pi_0}\frac{\partial^3 l}{\partial \pi_0 \partial \sigma^2 \partial \theta} \\
\frac{\partial S}{\partial \sigma^2} &= \frac{\partial^3 l}{\partial (\sigma^2)^3}\frac{\partial^2 l}{\partial \pi_0 \partial \theta} + \frac{\partial^2 l}{\partial (\sigma^2)^2}\frac{\partial^3 l}{\partial \pi_0 \partial \sigma^2 \partial \theta} - \frac{\partial^3 l}{\partial \pi_0 \partial (\sigma^2)^2}\frac{\partial^2 l}{\partial \sigma^2 \partial \theta} - \frac{\partial^2 l}{\partial \sigma^2 \partial \pi_0}\frac{\partial^2 l}{\partial (\sigma^2)^2 \partial \theta} \\
\frac{\partial S}{\partial \beta_{j\cdot}} &= \frac{\partial^3 l}{\partial (\sigma^2)^2 \partial \beta_{j\cdot}}\frac{\partial^2 l}{\partial \pi_0 \partial \theta} + \frac{\partial^2 l}{\partial (\sigma^2)^2}\frac{\partial^3 l}{\partial \pi_0 \partial \theta \partial \beta_{j\cdot}} - \frac{\partial^3 l}{\partial \sigma^2 \partial \pi_0 \partial \beta_{j\cdot}}\frac{\partial^2 l}{\partial \sigma^2 \partial \theta} - \frac{\partial^2 l}{\partial \sigma^2 \partial \pi_0}\frac{\partial^3 l}{\partial \sigma^2 \partial \theta \partial \beta_{j\cdot}}.
\end{aligned}$$

From Equation (6) we know that

$$\frac{\partial \hat{\pi}_0}{\partial \theta} = \Big(\frac{\partial^2 l}{\partial \sigma^2 \partial \pi_0}\frac{\partial^2 l}{\partial \sigma^2 \partial \theta} - \frac{\partial^2 l}{\partial (\sigma^2)^2}\frac{\partial^2 l}{\partial \pi_0 \partial \theta}\Big)\Big/\Big[\frac{\partial^2 l}{\partial \pi_0^2}\frac{\partial^2 l}{\partial (\sigma^2)^2} - (\frac{\partial^2 l}{\partial \pi_0 \partial \sigma^2})^2\Big]$$

$$\frac{\partial \hat{\sigma}^2}{\partial \theta} = \Big(\frac{\partial^2 l}{\partial \pi_0 \partial \sigma^2}\frac{\partial^2 l}{\partial \pi_0 \partial \theta} - \frac{\partial^2 l}{\partial \pi_0^2}\frac{\partial^2 l}{\partial \sigma^2 \partial \theta}\Big)\Big/\Big[\frac{\partial^2 l}{\partial \pi_0^2}\frac{\partial^2 l}{\partial (\sigma^2)^2} - (\frac{\partial^2 l}{\partial \pi_0 \partial \sigma^2})^2\Big]$$

$\hat{\pi}_0$ and $\hat{\sigma}^2$ are also functions of $\boldsymbol{\beta}_x$ and $\boldsymbol{\beta}_y$. Similarly,

$$\begin{pmatrix} \frac{\partial \hat{\pi}_0}{\partial \beta_{j\cdot}} \\ \frac{\partial \hat{\sigma}^2}{\partial \beta_{j\cdot}} \end{pmatrix} = -\begin{pmatrix} \frac{\partial^2 l}{\partial \pi_0^2} & \frac{\partial^2 l}{\partial \pi_0 \partial \sigma^2} \\ \frac{\partial^2 l}{\partial \sigma^2 \partial \pi_0} & \frac{\partial^2 l}{\partial (\sigma^2)^2} \end{pmatrix}^{-1} \begin{pmatrix} \frac{\partial^2 l}{\partial \pi_0 \partial \beta_{j\cdot}} \\ \frac{\partial^2 l}{\partial \sigma^2 \partial \beta_{j\cdot}} \end{pmatrix}.$$

Hence

$$\frac{\partial \hat{\pi}_0}{\partial \beta_{j\cdot}} = \Big(\frac{\partial^2 l}{\partial \pi_0 \partial \sigma^2}\frac{\partial^2 l}{\partial \sigma^2 \partial \beta_{j\cdot}} - \frac{\partial^2 l}{\partial (\sigma^2)^2}\frac{\partial^2 l}{\partial \pi_0 \partial \beta_{j\cdot}}\Big)\Big/\Big[\frac{\partial^2 l}{\partial \pi_0^2}\frac{\partial^2 l}{\partial (\sigma^2)^2} - (\frac{\partial^2 l}{\partial \pi_0 \partial \sigma^2})^2\Big]$$

$$\frac{\partial \hat{\sigma}^2}{\partial \beta_{j\cdot}} = \Big[\frac{\partial^2 l}{\partial \pi_0 \partial \sigma^2}\frac{\partial^2 l}{\partial \pi_0 \partial \beta_{j\cdot}} - \frac{\partial^2 l}{\partial \pi_0^2}\frac{\partial^2 l}{\partial \sigma^2 \partial \beta_{j\cdot}}\Big]\Big/\Big[\frac{\partial^2 l}{\partial \pi_0^2}\frac{\partial^2 l}{\partial (\sigma^2)^2} - (\frac{\partial^2 l}{\partial \pi_0 \partial \sigma^2})^2\Big].$$

To compute the partial derivatives of S, we need the second and third order partial derivatives of the log-likelihood l. The second order derivatives are:

$$\frac{\partial^2 l}{\partial (\sigma^2)^2} = (1-\pi_0)\sum_j \frac{\frac{\partial^2 f_2}{\partial (\sigma^2)^2} f - (1-\pi_0)\big(\frac{\partial f_2}{\partial \sigma^2}\big)^2}{f^2}\Big|_{(\beta_{jx},\beta_{jy},s_{jx},s_{jy};\theta,\pi_0,\sigma^2)}$$

$$\frac{\partial^2 l}{\partial \pi_0 \partial \theta} = \sum_j \frac{\frac{\partial f_1}{\partial \theta} f_2 - f_1 \frac{\partial f_2}{\partial \theta}}{f^2}\Big|_{(\beta_{jx},\beta_{jy},s_{jx},s_{jy};\theta,\pi_0,\sigma^2)}$$

$$\frac{\partial^2 l}{\partial \pi_0 \partial \sigma^2} = -\sum_j \frac{f_1 \frac{\partial f_2}{\partial \sigma^2}}{f^2}\Big|_{(\beta_{jx},\beta_{jy},s_{jx},s_{jy};\theta,\pi_0,\sigma^2)}$$

$$\frac{\partial^2 l}{\partial \sigma^2 \partial \theta} = (1-\pi_0)\sum_j \frac{\frac{\partial^2 f_2}{\partial \sigma^2 \partial \theta} f - \frac{\partial f_2}{\partial \sigma^2}\frac{\partial f}{\partial \theta}}{f^2}\Big|_{(\beta_{jx},\beta_{jy},s_{jx},s_{jy};\theta,\pi_0,\sigma^2)}$$

$$\frac{\partial^2 l}{\partial \pi_0 \partial \beta_{j\cdot}} = \frac{\frac{\partial f_1}{\partial \beta_{j\cdot}} f_2 - f_1 \frac{\partial f_2}{\partial \beta_{j\cdot}}}{f^2}\Big|_{(\beta_{jx},\beta_{jy},s_{jx},s_{jy};\theta,\pi_0,\sigma^2)}$$

$$\frac{\partial^2 l}{\partial \sigma^2 \partial \beta_{j\cdot}} = (1-\pi_0)\frac{\frac{\partial^2 f_2}{\partial \sigma^2 \partial \beta_{j\cdot}} f - \frac{\partial f_2}{\partial \sigma^2}\frac{\partial f}{\partial \beta_{j\cdot}}}{f^2}\Big|_{(\beta_{jx},\beta_{jy},s_{jx},s_{jy};\theta,\pi_0,\sigma^2)}.$$

The third order derivatives are:

$$
\begin{aligned}
\frac{\partial^3 l}{\partial(\sigma^2)^2\partial\theta} =&\sum_j \frac{1-\pi_0}{f^3}\Big\{\Big[\frac{\partial^3 f_2}{\partial(\sigma^2)^2\partial\theta}f+\frac{\partial^2 f_2}{\partial(\sigma^2)^2}\frac{\partial f}{\partial\theta}-2(1-\pi_0)\frac{\partial f_2}{\partial\sigma^2}\frac{\partial^2 f_2}{\partial\sigma^2\partial\theta}\Big]f\\
&-2\Big[\frac{\partial^2 f_2}{\partial(\sigma^2)^2}f-(1-\pi_0)\Big(\frac{\partial f_2}{\partial\sigma^2}\Big)^2\Big]\frac{\partial f}{\partial\theta}\Big\}\Big|_{(\beta_{jx},\beta_{jy},s_{jx},s_{jy};\theta,\pi_0,\sigma^2)}\\
\frac{\partial^3 l}{\partial\pi_0\partial\theta^2} =&\sum_j \frac{1}{f^3}\Big[\Big(\frac{\partial^2 f_1}{\partial\theta^2}f_2-f_1\frac{\partial^2 f_2}{\partial\theta^2}\Big)f-2\Big(\frac{\partial f_1}{\partial\theta}f_2-f_1\frac{\partial f_2}{\partial\theta}\Big)\frac{\partial f}{\partial\theta}\Big]\Big|_{(\beta_{jx},\beta_{jy},s_{jx},s_{jy};\theta,\pi_0,\sigma^2)}\\
\frac{\partial^3 l}{\partial\pi_0\partial\sigma^2\partial\theta} =&-\sum_j \frac{1}{f^3}\Big[\Big(\frac{\partial f_1}{\partial\theta}\frac{\partial f_2}{\partial\sigma^2}+f_1\frac{\partial^2 f_2}{\partial\sigma^2\partial\theta}\Big)f-2f_1\frac{\partial f_2}{\partial\sigma^2}\frac{\partial f}{\partial\theta}\Big]\Big|_{(\beta_{jx},\beta_{jy},s_{jx},s_{jy};\theta,\pi_0,\sigma^2)}\\
\frac{\partial^3 l}{\partial\sigma^2\partial\theta^2} =&\sum_j \frac{1-\pi_0}{f^3}\Big[\Big(\frac{\partial^3 f_2}{\partial\sigma^2\partial\theta^2}f-\frac{\partial f_2}{\partial\sigma^2}\frac{\partial^2 f}{\partial\theta^2}\Big)f-2\Big(\frac{\partial^2 f_2}{\partial\sigma^2\partial\theta}f-\frac{\partial f_2}{\partial\sigma^2}\frac{\partial f}{\partial\theta}\Big)\frac{\partial f}{\partial\theta}\Big]\Big|_{(\beta_{jx},\beta_{jy},s_{jx},s_{jy};\theta,\pi_0,\sigma^2)}\\
\frac{\partial^3 l}{\partial\pi_0\partial(\sigma^2)^2} =&-\sum_j \frac{1}{f^3}\Big[\Big(\frac{\partial f_1}{\partial\sigma^2}\frac{\partial f_2}{\partial\sigma^2}-f_1\frac{\partial^2 f_2}{\partial(\sigma^2)^2}\Big)f-2f_1\frac{\partial f_2}{\partial\sigma^2}\frac{\partial f}{\partial\sigma^2}\Big]\Big|_{(\beta_{jx},\beta_{jy},s_{jx},s_{jy};\theta,\pi_0,\sigma^2)}\\
\frac{\partial^3 l}{\partial\pi_0^2\partial\theta} =&-2\sum_j \frac{\Big(\frac{\partial f_1}{\partial\theta}f_2-f_1\frac{\partial f_2}{\partial\theta}\Big)(f_1-f_2)}{f^3}\Big|_{(\beta_{jx},\beta_{jy},s_{jx},s_{jy};\theta,\pi_0,\sigma^2)}\\
\frac{\partial^3 l}{\partial\pi_0^2\partial\sigma^2} =&2\sum_j \frac{f_1\frac{\partial f_2}{\partial\sigma^2}(f_1-f_2)}{f^3}\Big|_{(\beta_{jx},\beta_{jy},s_{jx},s_{jy};\theta,\pi_0,\sigma^2)}\\
\frac{\partial^3 l}{\partial(\sigma^2)^3} =&(1-\pi_0)\sum_j \frac{1}{f^3}\Big\{\Big[\frac{\partial^3 f_2}{\partial(\sigma^2)^3}f-(1-\pi_0)\frac{\partial f_2}{\partial\sigma^2}\frac{\partial^2 f_2}{\partial(\sigma^2)^2}\Big]f-2\Big[\frac{\partial^2 f_2}{\partial(\sigma^2)^2}f-(1-\pi_0)(\frac{\partial f_2}{\partial\sigma^2})^2\Big]\frac{\partial f_2}{\partial\sigma^2}\Big\}\Big|_{(\beta_{jx},\beta_{jy},s_{jx},s_{jy};\theta,\pi_0,\sigma^2)}
\end{aligned}
$$

Third order derivatives involving β_{jx} and β_{jy}:

$$
\begin{aligned}
\frac{\partial^3 l}{\partial(\sigma^2)^2\partial\beta_{j\cdot}} =&\frac{1-\pi_0}{f^3}\Big\{\Big[\frac{\partial^3 f_2}{\partial(\sigma^2)^2\partial\beta_{j\cdot}}f+\frac{\partial^2 f_2}{\partial(\sigma^2)^2}\frac{\partial f}{\partial\beta_{j\cdot}}-2(1-\pi_0)\frac{\partial f_2}{\partial\sigma^2}\frac{\partial^2 f_2}{\partial\sigma^2\partial\beta_{j\cdot}}\Big]f\\
&-2\Big[\frac{\partial^2 f_2}{\partial(\sigma^2)^2}f-(1-\pi_0)\Big(\frac{\partial f_2}{\partial\sigma^2}\Big)^2\Big]\frac{\partial f}{\partial\beta_{j\cdot}}\Big\}\Big|_{(\beta_{jx},\beta_{jy},s_{jx},s_{jy};\theta,\pi_0,\sigma^2)}\\
\frac{\partial^3 l}{\partial\pi_0\partial\theta\partial\beta_{j\cdot}} =&\frac{1}{f^3}\Big[\Big(\frac{\partial^2 f_1}{\partial\theta\partial\beta_{j\cdot}}f_2+\frac{\partial f_1}{\partial\theta}\frac{\partial f_2}{\partial\beta_{j\cdot}}-\frac{\partial f_1}{\partial\beta_{j\cdot}}\frac{\partial f_2}{\partial\theta}-f_1\frac{\partial^2 f_2}{\partial\theta\partial\beta_{j\cdot}}\Big)f\\
&-2\Big(\frac{\partial f_1}{\partial\theta}f_2-f_1\frac{\partial f_2}{\partial\theta}\Big)\frac{\partial f}{\partial\beta_{j\cdot}}\Big]\Big|_{(\beta_{jx},\beta_{jy},s_{jx},s_{jy};\theta,\pi_0,\sigma^2)}\\
\frac{\partial^3 l}{\partial\pi_0\partial\sigma^2\partial\beta_{j\cdot}} =&-\frac{1}{f^3}\Big[\Big(\frac{\partial f_1}{\partial\beta_{j\cdot}}\frac{\partial f_2}{\partial\sigma^2}+f_1\frac{\partial^2 f_2}{\partial\sigma^2\partial\beta_{j\cdot}}\Big)f-2f_1\frac{\partial f_2}{\partial\sigma^2}\frac{\partial f}{\partial\beta_{j\cdot}}\Big]\Big|_{(\beta_{jx},\beta_{jy},s_{jx},s_{jy};\theta,\pi_0,\sigma^2)}\\
\frac{\partial^3 l}{\partial\sigma^2\partial\theta\partial\beta_{j\cdot}} =&\frac{1-\pi_0}{f^3}\Big[\Big(\frac{\partial^3 f_2}{\partial\sigma^2\partial\theta\partial\beta_{j\cdot}}f+\frac{\partial^2 f_2}{\partial\sigma^2\partial\theta}\frac{\partial f}{\partial\beta_{j\cdot}}-\frac{\partial^2 f_2}{\partial\sigma^2\partial\beta_{j\cdot}}\frac{\partial f}{\partial\theta}-\frac{\partial f_2}{\partial\sigma^2}\frac{\partial^2 f}{\partial\theta\partial\beta_{j\cdot}}\Big)f\\
&-2\Big(\frac{\partial^2 f_2}{\partial\sigma^2\partial\theta}f-\frac{\partial f_2}{\partial\sigma^2}\frac{\partial f}{\partial\theta}\Big)\frac{\partial f}{\partial\beta_{j\cdot}}\Big]\Big|_{(\beta_{jx},\beta_{jy},s_{jx},s_{jy};\theta,\pi_0,\sigma^2)}.
\end{aligned}
$$

The partial derivatives of $f_1(\beta_x,\beta_y,s_x,s_y;\theta)$, $f_2(\beta_x,\beta_y,s_x,s_y;\theta,\sigma^2)$ and $f(\beta_x,\beta_y,s_x,s_y;\theta,\pi_0,\sigma^2)$ can be derived from Equations (1)-(3) and hence are omitted here.

3 Summary of existing methods of Mendelian randomization

Inverse-variance weighted method (IVW): The IVW aggregates the ratio estimators by taking a linear combination, weighted by the inverse of their variances [7]. For SNP j, denote by $\hat{\beta}_{jx}$ the GWAS estimate for the exposure X, by $\hat{\beta}_{jy}$ the GWAS estimate for the outcome Y, and by $\hat{\theta}_j$ the corresponding ratio estimator. Let s_{jy} be the standard error corresponding to $\hat{\beta}_{jy}$. The IVW estimator is define by

$$\hat{\theta}_{IVW} = \frac{\sum_j \hat{\beta}_{jx}^2 s_{jy}^{-2} \hat{\theta}_j}{\sum_j \hat{\beta}_{jx}^2 s_{jy}^{-2}}$$

Here $\hat{\beta}_{jx}^{-2} s_{jy}^2$ is an approximation of the variance of $\hat{\theta}_j$, assuming that the variance of $\hat{\beta}_{jx}$ is small enough to be ignored.

Weighted median: The simple median estimator is the median of the ratio estimators, with equal weight on each variant. The weighted median method assigns weights w_j to ratio estimators to improve efficiency. The weight w_j is proportional to the inverse variance of $\hat{\theta}_j$ and is normalized to sum to 1, i.e.

$$w_j' = \hat{\beta}_{jx}^2 s_{jy}^{-2}, \quad w_j = \frac{w_j'}{\sum_j w_j'}.$$

The estimator is defined by taking median of the $\hat{\theta}_j$'s based on the weighted distribution [4].

Weighted mode: The weighted mode takes the mode of the estimated distribution of the ratio estimators, weighted by the same w_j as the weighted median. This estimator is consistent under the ZEro Modal Pleiotropy Assumption (ZEMPA) [8].

Egger regression: Egger regression fits a linear model $\hat{\beta}_y = \theta_0 + \theta \hat{\beta}_x$ and uses the regression slope $\hat{\theta}$ as estimate of the causal effect and the intercept $\hat{\theta}_0$ to quantify directional pleiotropy [3]. This method gives unbiased causal estimates in the presence of directional pleiotropy but requires the InSIDE assumption.

LD score regression: Following notations in the main manuscript, let (u_x, u_y) denote the direct effects and (β_x, β_y) denote the total effects. Let θ be the causal

effect. And it holds that

$$\beta_x = u_x, \quad \beta_y = u_y + \theta u_x.$$

If the InSIDE assumption $cov(u_x, u_y) = 0$ holds across all SNPs, it follows that $cov(\beta_x, \beta_y) = cov(u_x, u_y) + \theta var(\beta_x)$ and $\theta = \frac{cov(\beta_y, \beta_x)}{var(\beta_x)} = \frac{\rho_g}{h_x^2}$, where ρ_g is the genetic covariance between X and Y and h_x^2 is the heritability of X. LD score regression can estimate both ρ_g and h_x^2, hence can estimate the causal effect θ [6, 5]. However, the InSIDE assumption is unlikely to hold across all SNPs since two traits can share common genetic pathways. We still report the results since it characterizes the effects purely based on genetic correlation.

The LD score regression estimator ρ_g/h_x^2 is nearly equivalent to Egger regression using the same set of SNPs. Under large sample size, Egger regression estimate is approximately

$$\hat{\theta}_{Egger} \approx \frac{cov(\hat{\beta}_y, \hat{\beta}_x)}{var(\hat{\beta}_x)} \approx \frac{cov(\hat{\beta}_y, \beta_x)}{var(\beta_x)} = \frac{cov(\beta_y, \beta_x)}{var(\beta_x)}.$$

The last equation uses the fact the that error term of $\hat{\beta}_y$ is independent of β_x. We can see that both methods estimate the same quantity, though using different techniques.

4 Additional simulation settings

4.1 Allowing for SNPs of distinctly larger effects

In Methods section, we describe the basic simulation setting where direct effect sizes of 200,000 independent SNPs are generated from the 4-component model (**Figure 1a**) with parameters $\pi_1, \pi_2, \pi_3, \sigma_x^2, \sigma_y^2, \sigma_{xy}$. Here we describe a scenario (*Scenario B*) that is adapted from *Scenario A* by allowing a small number of SNPs to have distinctly larger effects than others and those SNPs are more likely to be valid IVs.

We now explain *Scenario B* by elaborating the changes to each mixture component (**Figure 1a**) compared to *Scenario A*.

- Component 1: Randomly draw 60 SNPs from this component to assign large X-effects by $u_x \sim N(0, 10\sigma_x^2)$; for the rest of SNPs in this component, simulate u_x from $N(0, \sigma_x^2)$ - these are the SNPs with smaller effects on X.

- Component 2: Randomly draw 20 SNPs from this component to assign large effects for X (SNP set 2.x); and independently draw 20 SNPs to assign large effects for Y (SNP set 2.y). If 2.x and 2.y overlap (which is rare), we generate effects sizes of the overlapping SNPs by $\binom{u_x}{u_y} \sim N\left(\binom{0}{0}, 10\begin{pmatrix} \sigma_x^2 & \sigma_{xy} \\ \sigma_{xy} & \sigma_y^2 \end{pmatrix}\right)$; for SNPs in 2.x but not in 2.y, generate effect sizes by $\binom{u_x}{u_y} \sim N\left(\binom{0}{0}, \begin{pmatrix} 10\sigma_x^2 & \sqrt{10}\sigma_{xy} \\ \sqrt{10}\sigma_{xy} & \sigma_y^2 \end{pmatrix}\right)$; for SNPs in 2.y but not in 2.x, generate effect sizes by $\binom{u_x}{u_y} \sim N\left(\binom{0}{0}, \begin{pmatrix} \sigma_x^2 & \sqrt{10}\sigma_{xy} \\ \sqrt{10}\sigma_{xy} & 10\sigma_y^2 \end{pmatrix}\right)$; for the rest of the SNPs in this component, assign effects by $\binom{u_x}{u_y} \sim N\left(\binom{0}{0}, \begin{pmatrix} \sigma_x^2 & \sigma_{xy} \\ \sigma_{xy} & \sigma_y^2 \end{pmatrix}\right)$ - these are the SNPs with smaller effects on both X and Y.

- Component 3: Randomly draw 60 SNPs from this component to assign large Y-effects by $u_y \sim N(0, 10\sigma_y^2)$; for the rest of SNPs in this component, simulate u_y from $N(0, \sigma_y^2)$ - these are the SNPs with smaller effects on Y.

- Component 4: Zero effects for both X and Y.

Two sets of parameter values are considered analogous to *Scenario A*:

- (B.1) 50% causal SNPs for X are valid IVs: $\pi_1 = \pi_2 = 0.01$.

- (B.2) 25% causal SNPs for X are valid IVs: $\pi_1 = 0.005, \pi_2 = 0.015$.

For both scenarios, we set $\pi_3 = 0.01$, $\pi_4 = 0.97$, $\sigma_x^2 = \sigma_y^2 = 5 \times 10^{-5}$, $\sigma_{xy} = 0.5\sigma_x\sigma_y$.

Take *Scenario (B.1)* as an example. There are 2,000 SNPs in each of Component 1-3. There are 80 (0.04% of the total 200K) SNPs of larger effects among the 4,000 causal SNPs for X and 3/4 of them are valid IVs; however, among the 3,920 SNPs of smaller effects, only around 1/2 are valid IVs. This holds similarly for Y. *Scenario B* is closer to the assumptions made by existing MR methods.

4.2 Non-normal effect size

We conduct simulations to study the performance of MRMix under non-normally distributed effect sizes. The effect sizes are still generated from the four-component model (**Figure 1a**), but follow Laplace and T distributions instead of normal distribution.

4.2.1 Laplace distribution

For components 1 and 3, we simulate effect sizes from univariate Laplace distribution centered at 0 with variance σ_x^2 and σ_y^2 respectively, which have density

$$f_1(x) = \frac{1}{\sqrt{2}\sigma_x}\exp(-\sqrt{2}|x|/\sigma_x), \quad f_3(y) = \frac{1}{\sqrt{2}\sigma_y}\exp(-\sqrt{2}|y|/\sigma_y).$$

For component 2, we use bivariate Laplace distribution with mean $(0,0)$ and covariance matrix $\left(\begin{smallmatrix}\sigma_x^2 & \sigma_{xy}\\ \sigma_{xy} & \sigma_y^2\end{smallmatrix}\right)$, which has density

$$f_L(x,y) = \frac{1}{\pi\sigma_x\sigma_y\sqrt{1-\rho^2}}K_0\left(\sqrt{\frac{2\left(\frac{x^2}{\sigma_x^2} - \frac{2\rho xy}{\sigma_x\sigma_y} + \frac{y^2}{\sigma_y^2}\right)}{1-\rho^2}}\right)$$

where $\rho = \frac{\sigma_{xy}}{\sigma_x\sigma_y}$ and $K_0(\cdot)$ is the modified Bessel function of the second kind [2].

4.2.2 T distribution

For components 1 and 3, we simulate effect size from scaled univariate T distributions - $\sigma_x T_{20}$ and $\sigma_y T_{20}$ respectively. Here T_{20} is the standard T distribution with 20 degrees of freedom. For component 2, we use bivariate T distribution centered at $(0,0)$ and shape matrix $\left(\begin{smallmatrix}\sigma_x^2 & \sigma_{xy}\\ \sigma_{xy} & \sigma_y^2\end{smallmatrix}\right)$, which has density[1].

$$f_T(x,y) = \frac{\Gamma[(\nu+2)/2]}{\Gamma[\nu/2]\nu^{p/2}\pi^{p/2}\sigma_x\sigma_y\sqrt{1-\rho^2}}\left[1 + \frac{1}{\nu}\frac{\left(\frac{x^2}{\sigma_x^2} - \frac{2\rho xy}{\sigma_x\sigma_y} + \frac{y^2}{\sigma_y^2}\right)}{1-\rho^2}\right].$$

where $\Gamma(\cdot)$ is the Gamma function, $\rho = \frac{\sigma_{xy}}{\sigma_x\sigma_y}$ and ν is the degrees of freedom. We set $\nu = 20$.

Supplementary notes references

[1] Wikipedia - multivariate t-distribution. https://en.wikipedia.org/wiki/Multivariate_t-distribution, 11 2017.

[2] Wikipedia - multivariate laplace distribution. https://en.wikipedia.org/wiki/Multivariate_Laplace_distribution, 10 2018.

[3] Jack Bowden, George Davey Smith, and Stephen Burgess. Mendelian randomization with invalid instruments: effect estimation and bias detection through egger regression. *International journal of epidemiology*, 44(2):512–525, 2015.

[4] Jack Bowden, George Davey Smith, Philip C Haycock, and Stephen Burgess. Consistent estimation in mendelian randomization with some invalid instruments using a weighted median estimator. *Genetic epidemiology*, 40(4):304–314, 2016.

[5] Brendan Bulik-Sullivan, Hilary K Finucane, Verneri Anttila, Alexander Gusev, Felix R Day, Po-Ru Loh, Laramie Duncan, John RB Perry, Nick Patterson, and Elise B Robinson. An atlas of genetic correlations across human diseases and traits. *Nature genetics*, 47(11):1236, 2015.

[6] Brendan K Bulik-Sullivan, Po-Ru Loh, Hilary K Finucane, Stephan Ripke, Jian Yang, Nick Patterson, Mark J Daly, Alkes L Price, Benjamin M Neale, and Schizophrenia Working Group of the Psychiatric Genomics Consortium. LD score regression distinguishes confounding from polygenicity in genome-wide association studies. *Nature genetics*, 47(3):291, 2015.

[7] Stephen Burgess, Adam Butterworth, and Simon G Thompson. Mendelian randomization analysis with multiple genetic variants using summarized data. *Genetic epidemiology*, 37(7):658–665, 2013.

[8] Fernando Pires Hartwig, George Davey Smith, and Jack Bowden. Robust inference in summary data mendelian randomization via the zero modal

pleiotropy assumption. *International journal of epidemiology*, 46(6):1985–1998, 2017.

Supplementary Figures

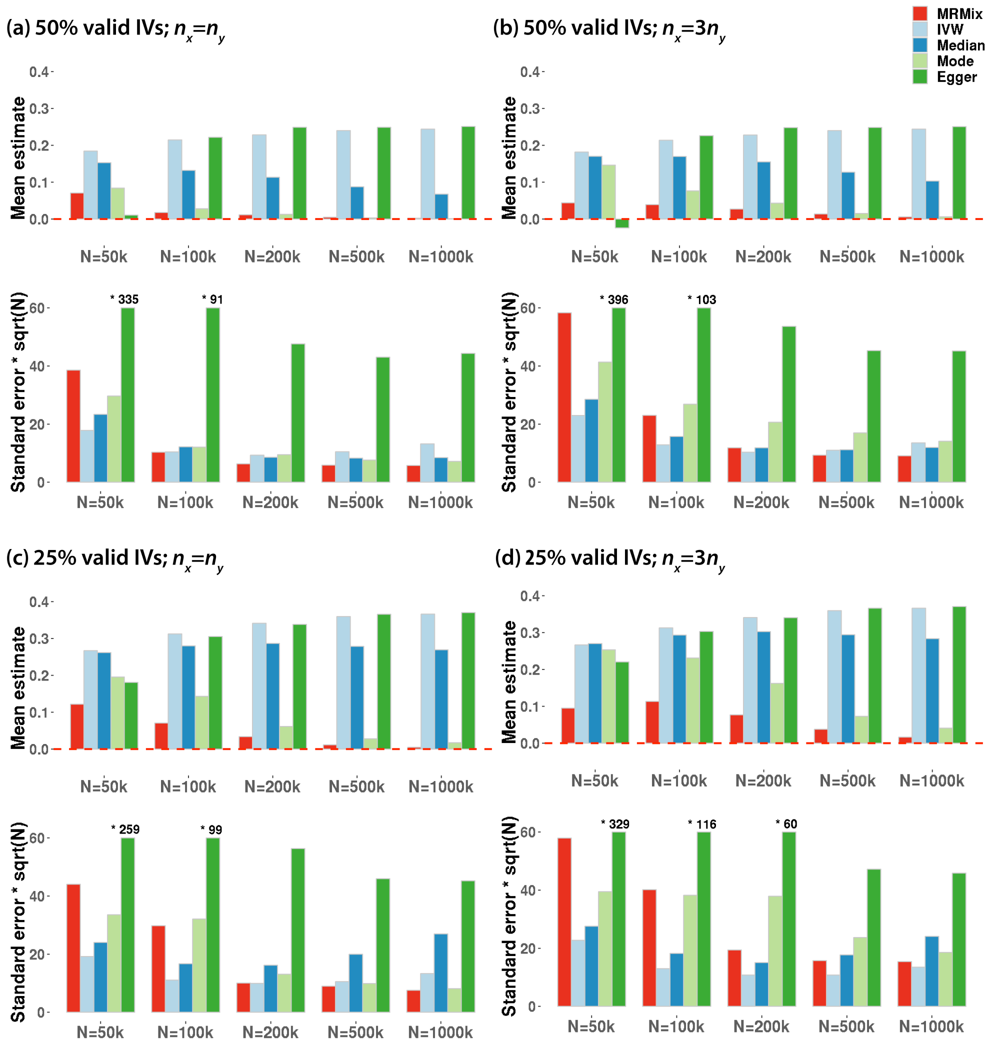

Supplementary Figure 1. Performance of MRMix and alternative methods for estimation of causal effects (θ) in simulation studies under <u>null causal effects</u>. The true effect θ is 0. Estimates of association coefficient for SNPs across two traits are simulated assuming an underlying four-component model for effect-size distribution (Scenario A, see **Methods**), where SNPs could have direct effects on neither traits, only on X, only on Y, or on both with the effects being correlated. The proportion of valid instruments, i.e. the SNPs which have only direct effects on X as a proportion of the total number of SNPs which are associated with X, are fixed at 50% or 25%. Mean (standard deviation) of causal estimates are reported over 100 simulations. $n_x = N$: sample size of the study associated with X; n_y: sample size of the study associated with Y. Standard error bars higher than 60 are truncated and marked with *true-value. The average number of IVs, defined as the SNPs which reach genome-wide significance (z-test $p < 5\times10^{-8}$) in the study associated with X, is 14, 105, 399, 1135 and 1780 for N=50k, 100k, 200k, 500k and 1000k, respectively. Source data are provided as a Source Data file.

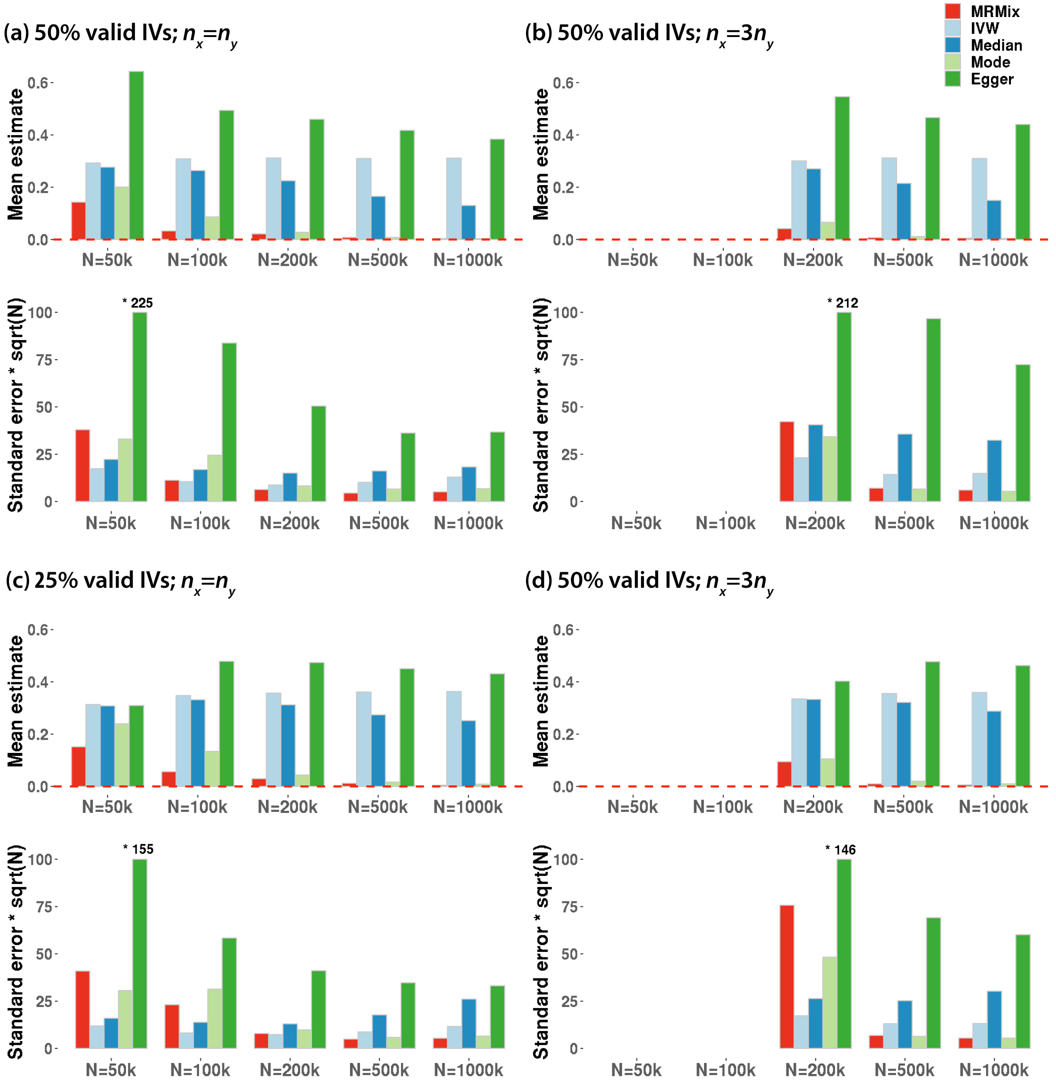

Supplementary Figure 2. Reverse directional analysis of MRMix and alternative methods in simulation studies where the <u>genetic correlation due to causal effect and pleiotropic effect are in the same direction</u>. Reported are the estimated causal effects of Y on X (true value=0) under the same scenarios as **Figure 2**. Estimates of association coefficient for SNPs across two traits are simulated assuming an underlying four-component model for effect-size distribution (Scenario A, see **Methods**), where SNPs could have direct effects on neither traits, only on X, only on Y, or on both with the effects being correlated. The proportion of valid instruments, i.e. the SNPs which have only direct effects on X as a proportion of the total number of SNPs which are associated with X, are fixed at 50% or 25%. Mean (standard deviation) of causal estimates are reported over 100 simulations. The true causal effect of X on Y is 0.2. $n_x = N$: sample size of the study associated with X; n_y: sample size of the study associated with Y. Standard error bars higher than 100 are truncated and marked with *true-value. Results for N=50K or 100K and $n_x = 3n_y$ are missing due to insufficient number of instruments (often ≤ 2). Source data are provided as a Source Data file.

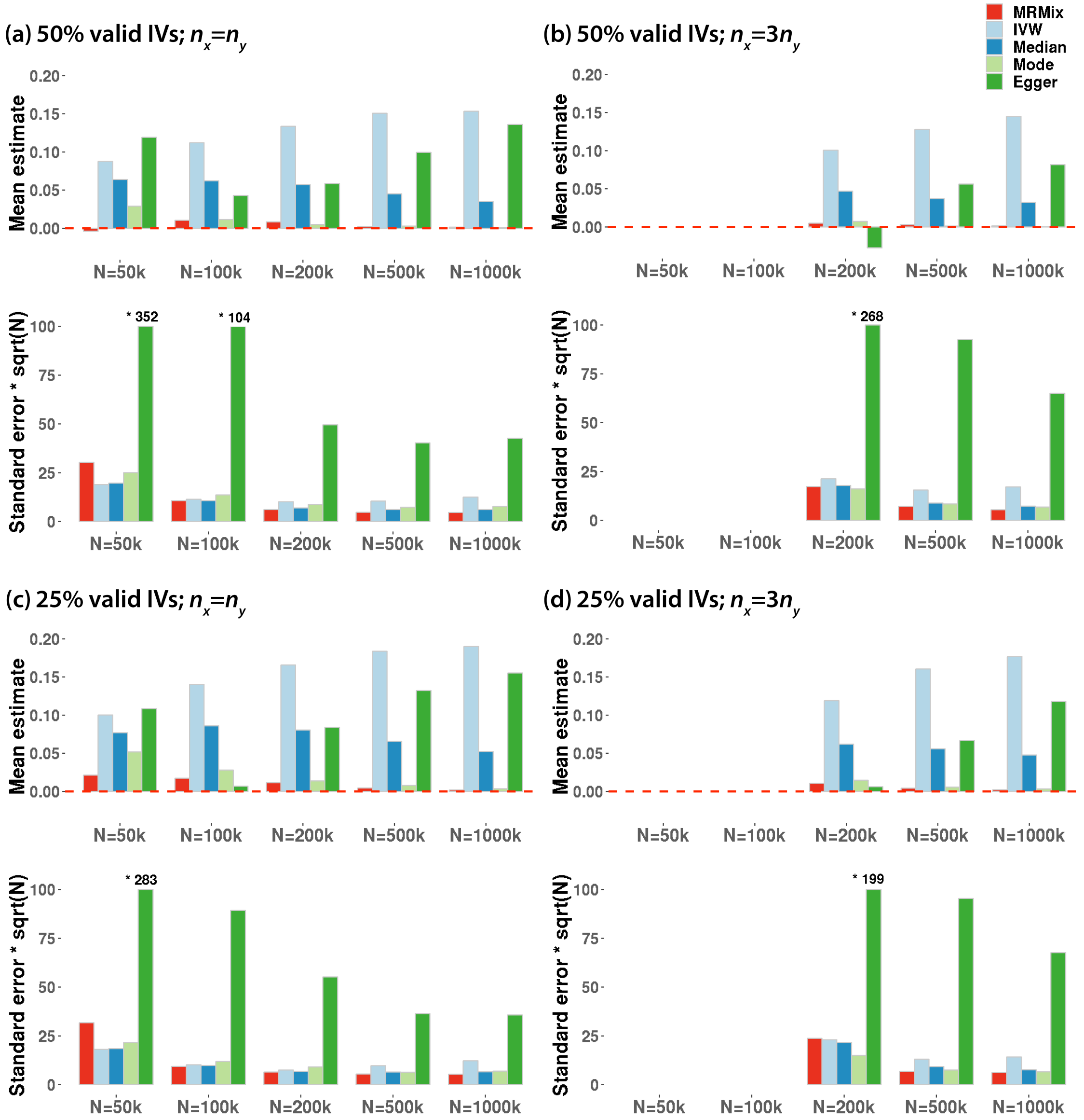

Supplementary Figure 3. Reverse directional analysis of MRMix and alternative methods in simulation studies where the <u>genetic correlation due to causal effect and pleiotropic effect are in opposite directions</u>. Reported are the estimated causal effects of Y on X (true value=0) under the same scenarios as **Figure 3**. Estimates of association coefficient for SNPs across two traits are simulated assuming an underlying four-component model for effect-size distribution (Scenario A, see **Methods**), where SNPs could have direct effects on neither traits, only on X, only on Y, or on both with the effects being correlated. The proportion of valid instruments, i.e. the SNPs which have only direct effects on X as a proportion of the total number of SNPs which are associated with X, are fixed at 50% or 25%. Mean (standard deviation) of causal estimates are reported over 100 simulations. The true causal effect of X on Y is -0.2. $n_x = N$: sample size of the study associated with X; n_y: sample size of the study associated with Y. Standard error bars higher than 100 are truncated and marked with *true-value. Results for N=50K or 100K and $n_x = 3n_y$ are missing due to insufficient number of instruments (often ≤ 2). Source data are provided as a Source Data file.

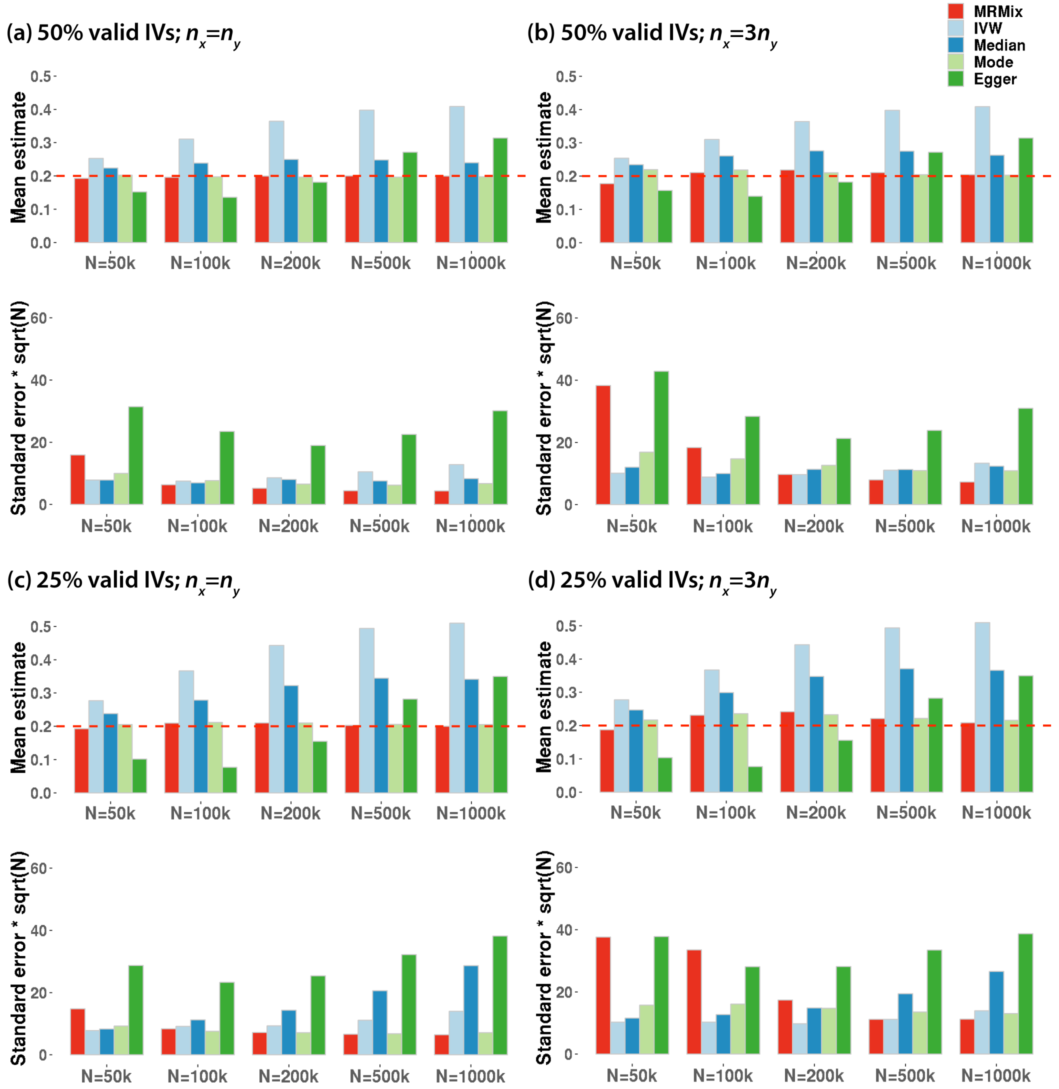

Supplementary Figure 4. Performance of MRMix and alternative methods for estimation of causal effects (θ) in simulation studies with <u>0.04% SNPs of distinctly larger effects</u> than others and <u>genetic correlation due to causal effect and pleiotropic effect in the same direction</u>. The true causal effect $\theta = 0.2$. Estimates of association coefficient for SNPs across two traits are simulated assuming an underlying four-component model for effect-size distribution for most SNPs, while allowing a small number of SNPs to have distinctly larger effects (Scenario B, see **Methods** and **Supplementary Notes Section 4.1**). SNPs could have direct effects on neither traits, only on X, only on Y, or on both with the effects being correlated. The proportion of valid instruments, i.e. the SNPs which have only direct effects on X as a proportion of the total number of SNPs which are associated with X, are fixed at 50% or 25%. Mean (standard deviation) of causal estimates are reported over 100 simulations. $n_x = N$: sample size of the study associated with X; n_y: sample size of the study associated with Y. The average number of IVs, defined as the SNPs which reach genome-wide significance (z-test $p < 5\times10^{-8}$) in the study associated with X, is 37, 138, 439, 1175 and 1809 for N=50k, 100k, 200k, 500k and 1000k, respectively. Source data are provided as a Source Data file.

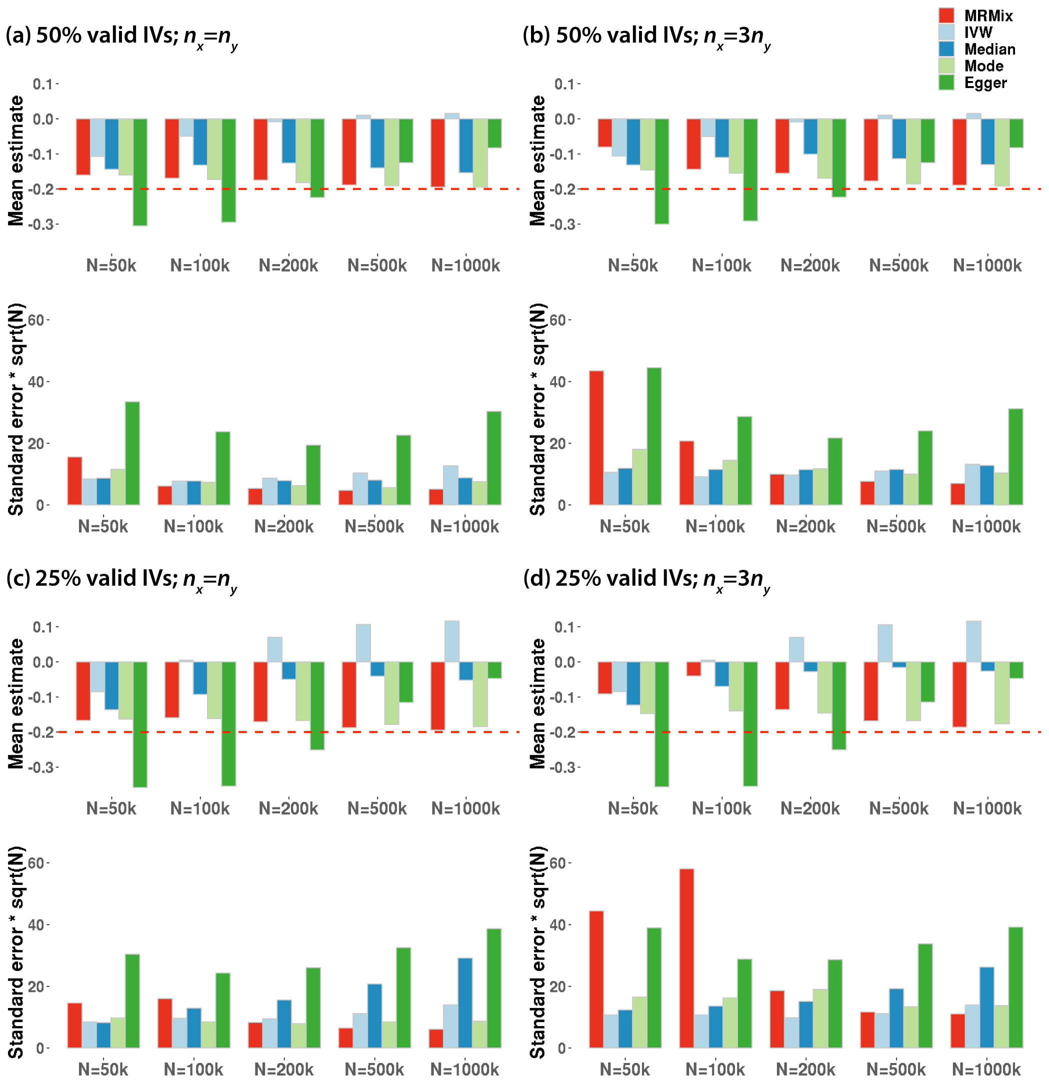

Supplementary Figure 5. Performance of MRMix and alternative methods for estimation of causal effects (θ) in simulation studies where <u>0.04% SNPs have distinctly larger effects</u> than others and <u>genetic correlation due to causal effect and pleiotropic effect are in opposite directions</u>. The true causal effect $\theta = -0.2$. Estimates of association coefficient for SNPs across two traits are simulated assuming an underlying four-component model for effect-size distribution for most SNPs, while allowing a small number of SNPs to have distinctly larger effects (Scenario B, see **Methods** and **Supplementary Notes Section 4.1**). SNPs could have direct effects on neither traits, only on X, only on Y, or on both with the effects being correlated. The proportion of valid instruments, i.e. the SNPs which have only direct effects on X as a proportion of the total number of SNPs which are associated with X, are fixed at 50% or 25%. Mean (standard deviation) of causal estimates are reported over 100 simulations. $n_x = N$: sample size of the study associated with X; n_y: sample size of the study associated with Y. The average number of IVs, defined as the SNPs which reach genome-wide significance (z-test $p < 5\times10^{-8}$) in the study associated with X, is 37, 138, 439, 1175 and 1809 for N=50k, 100k, 200k, 500k and 1000k, respectively. Source data are provided as a Source Data file.

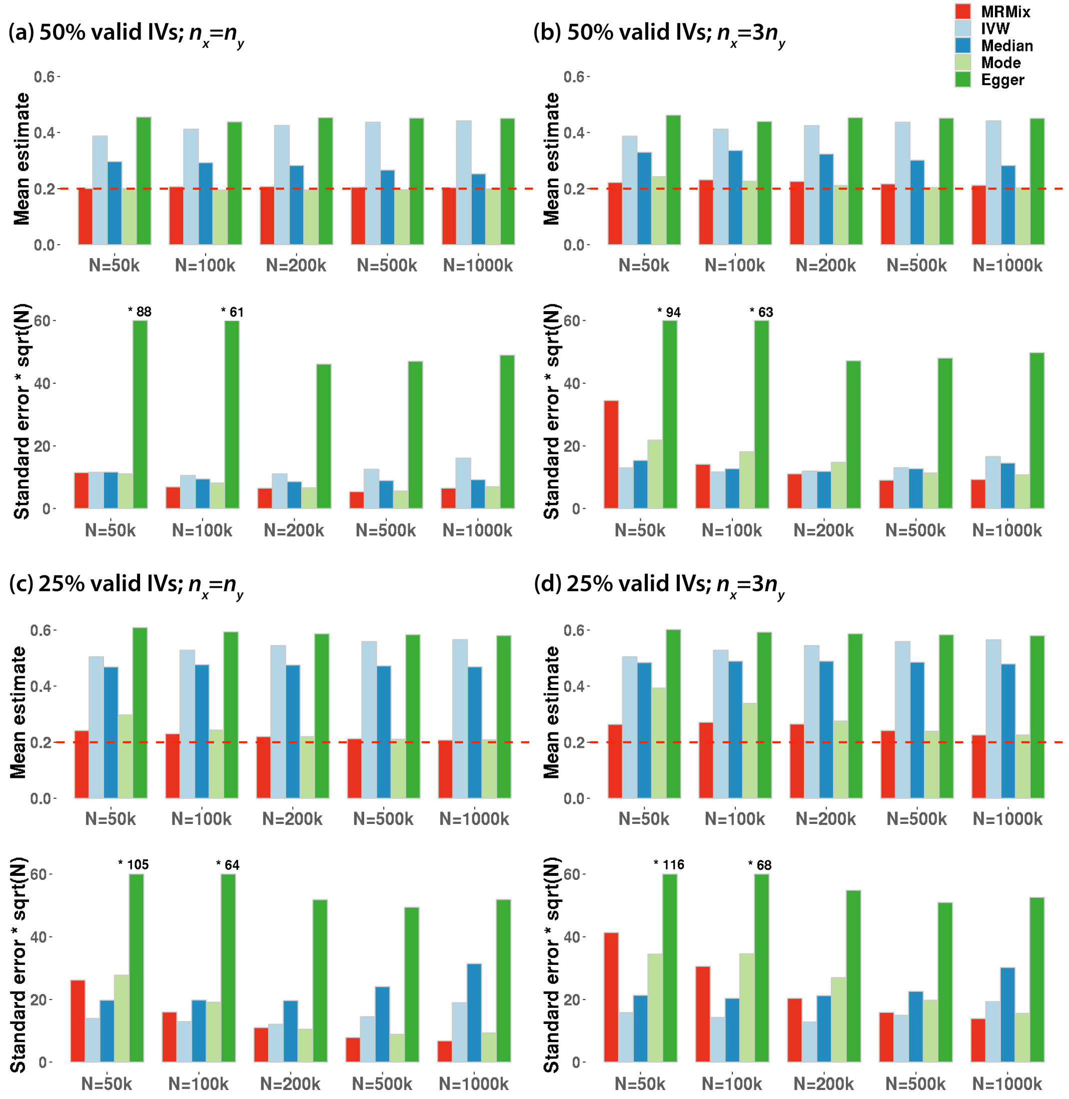

Supplementary Figure 6. Performance of MRMix and alternative methods for estimation of causal effects (θ) in simulation studies where effect sizes are generated from <u>Laplace distribution with genetic correlation due to causal effect and pleiotropic effect in the same direction</u>. The true causal effect $\theta = 0.2$. Estimates of association coefficient for SNPs across two traits are simulated assuming an underlying four-component model for effect-size distribution (Scenario C, see **Methods**), where SNPs could have direct effects on neither traits, only on X, only on Y, or on both with the effects being correlated. The proportion of valid instruments, i.e. the SNPs which have only direct effects on X, as a proportion of the total number of SNPs which are associated with X, are fixed at 50% or 25%. Mean (standard deviation) of causal estimates are reported over 100 simulations. $n_x = N$: sample size of the study associated with X; n_y: sample size of the study associated with Y. Standard error bars higher than 60 are truncated and marked with *true-value. The average number of IVs, defined as the SNPs which reach genome-wide significance (z-test $p < 5\times10^{-8}$) in the study associated with X, is 46, 156, 387, 888 and 1368 for N=50k, 100k, 200k, 500k and 1000k, respectively. Source data are provided as a Source Data file.

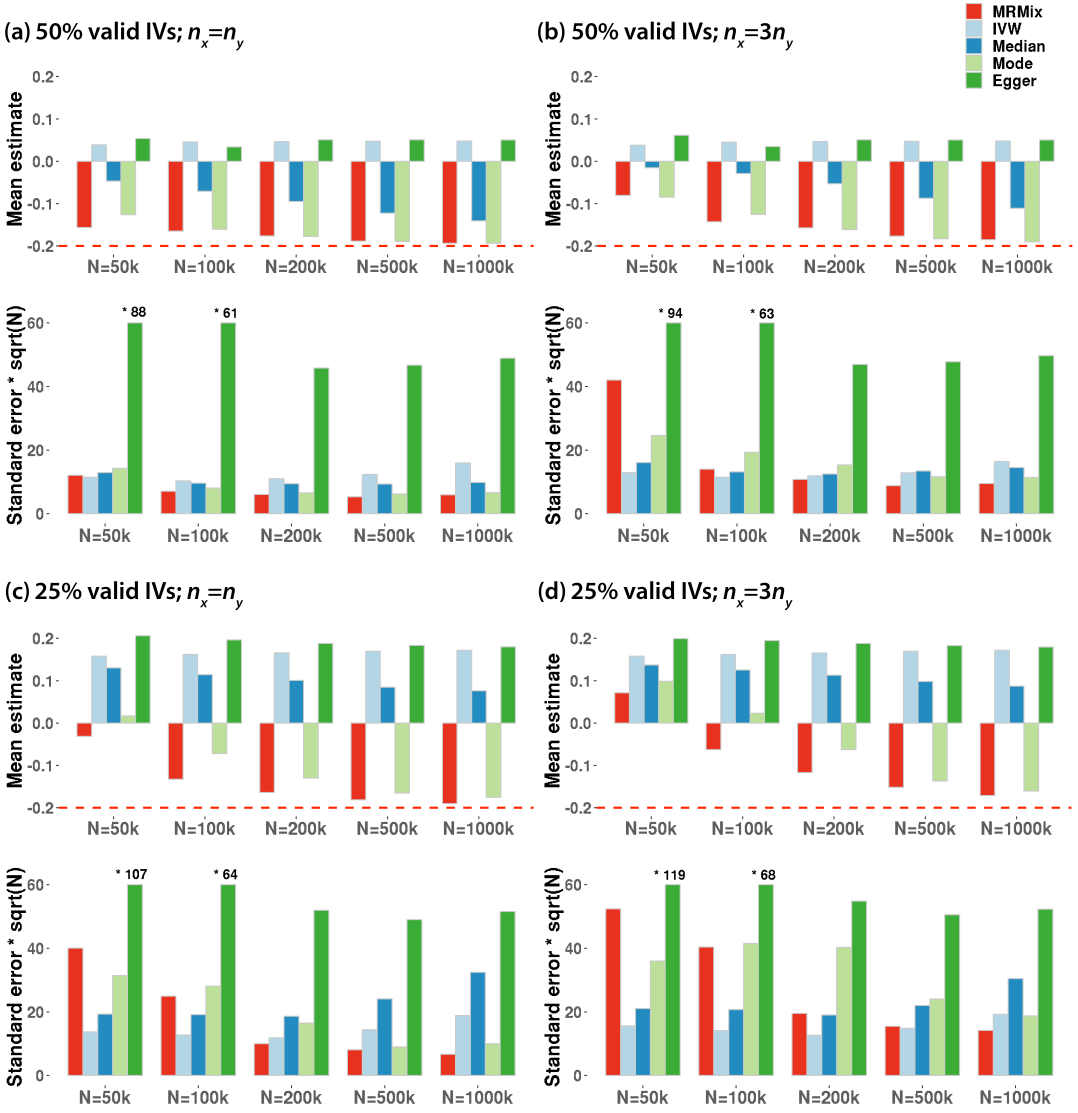

Supplementary Figure 7. Performance of MRMix and alternative methods for estimation of causal effects (θ) in simulation studies where effect sizes follow <u>Laplace distribution with genetic correlation due to causal effect and pleiotropic effect in opposite directions</u>. The true causal effect $\theta = -0.2$. Estimates of association coefficient for SNPs across two traits are simulated assuming an underlying four-component model for effect-size distribution (Scenario C, see **Methods**), where SNPs could have direct effects on neither traits, only on X, only on Y, or on both with the effects being correlated. The proportion of valid instruments, i.e. the SNPs which have only direct effects on X, as a proportion of the total number of SNPs which are associated with X, are fixed at 50% or 25%. Mean (standard deviation) of causal estimates are reported over 100 simulations. $n_x = N$: sample size of the study associated with X; n_y: sample size of the study associated with Y. Standard error bars higher than 60 are truncated and marked with *true-value. The average number of IVs, defined as the SNPs which reach genome-wide significance (z-test $p < 5\times10^{-8}$) in the study associated with X, is 46, 156, 387, 888 and 1368 for N=50k, 100k, 200k, 500k and 1000k, respectively. Source data are provided as a Source Data file.

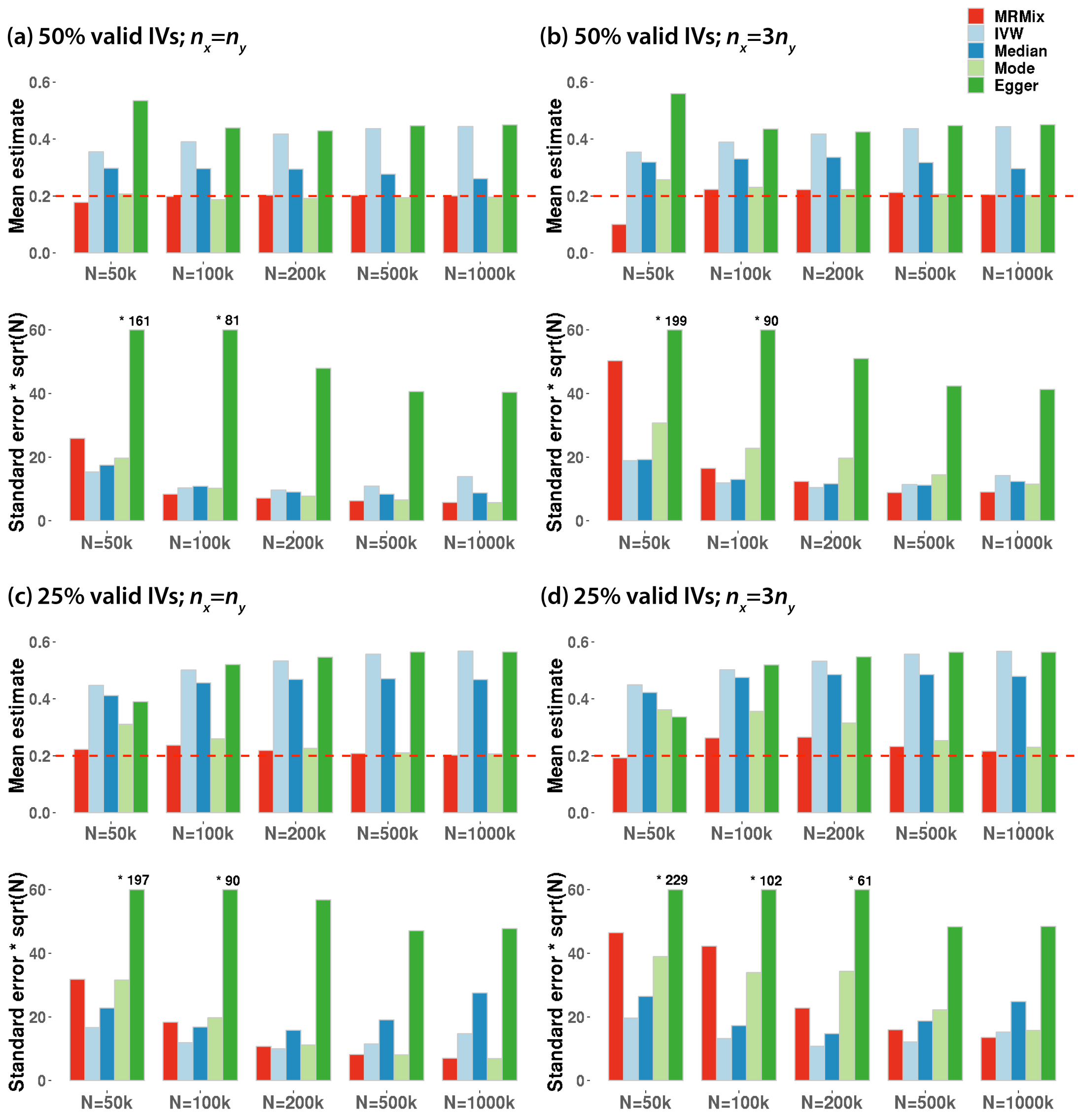

Supplementary Figure 8. Performance of MRMix and alternative methods for estimation of causal effects (θ) in simulation studies where effect sizes are generated from T distribution with genetic correlation due to causal effect and pleiotropic effect in the same direction. The true causal effect $\theta = 0.2$. Estimates of association coefficient for SNPs across two traits are simulated assuming an underlying four-component model for effect-size distribution (Scenario C, see **Methods**), where SNPs could have direct effects on neither traits, only on X, only on Y, or on both with the effects being correlated. The proportion of valid instruments, i.e. the SNPs which have only direct effects on X, as a proportion of the total number of SNPs which are associated with X, are fixed at 50% or 25%. Mean (standard deviation) of causal estimates are reported over 100 simulations. $n_x = N$: sample size of the study associated with X; n_y: sample size of the study associated with Y. Standard error bars higher than 60 are truncated and marked with *true-value. The average number of IVs, defined as the SNPs which reach genome-wide significance (z-test $p < 5{\times}10^{-8}$) in the study associated with X, is 27, 142, 458, 1191 and 1816 for N=50k, 100k, 200k, 500k and 1000k, respectively. Source data are provided as a Source Data file.

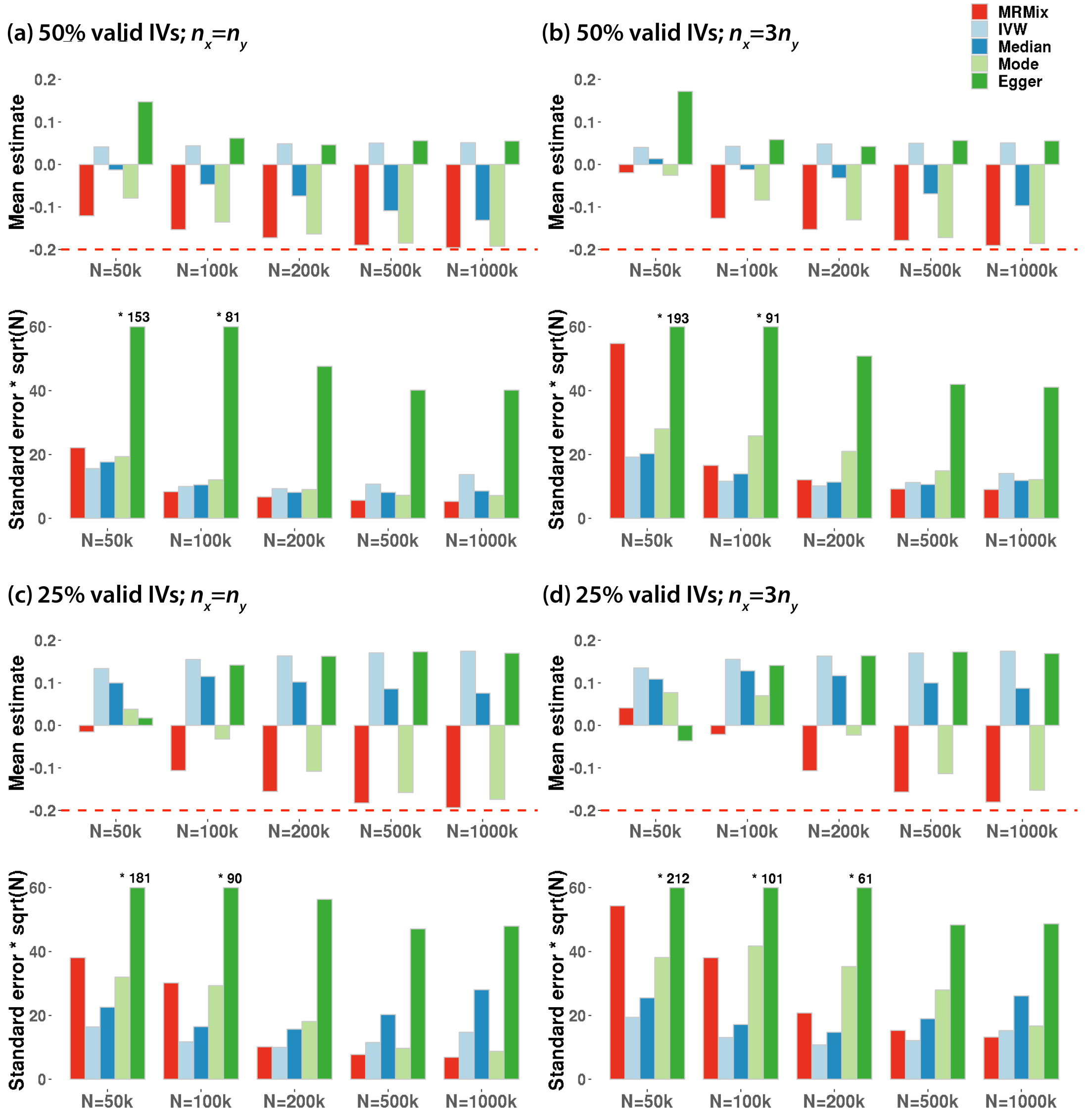

Supplementary Figure 9. Performance of MRMix and alternative methods for estimation of causal effects (θ) in simulation studies where effect sizes <u>follow T distribution with genetic correlation due to causal effect and pleiotropic effect in opposite directions</u>. The true causal effect $\theta = -0.2$. Estimates of association coefficient for SNPs across two traits are simulated assuming an underlying four-component model for effect-size distribution (Scenario C, see **Methods**), where SNPs could have direct effects on neither traits, only on X, only on Y, or on both with the effects being correlated. The proportion of valid instruments, i.e. the SNPs which have only direct effects on X, as a proportion of the total number of SNPs which are associated with X, are fixed at 50% or 25%. Mean (standard deviation) of causal estimates are reported over 100 simulations. $n_x = N$: sample size of the study associated with X; n_y: sample size of the study associated with Y. Standard error bars higher than 60 are truncated and marked with *true-value. The average number of IVs, defined as the SNPs which reach genome-wide significance (z-test $p < 5\times10^{-8}$) in the study associated with X, is 27, 142, 458, 1191 and 1816 for N=50k, 100k, 200k, 500k and 1000k, respectively. Source data are provided as a Source Data file.

Supplementary Tables

Supplementary Table 1. Simulation studies showing accuracy of asymptotic standard error estimate. Estimates of association coefficient for SNPs across two traits are simulated assuming an underlying four-component model for effect-size distribution (Scenario A, see **Methods**), where SNPs could have direct effects on neither traits, only on X, only on Y, or on both with the effects being correlated. The proportion of valid instruments, i.e. the SNPs which have only direct effects on X as a proportion of the total number of SNPs which are associated with X, are fixed at 50% or 25%. Mean of causal effect and standard error estimates are reported over 500 simulations. Source data are provided as a Source Data file.

Settings	$\boldsymbol{n_x}$ [a]	Average # of IVs (SD) [b]	Mean estimate	Empirical SE [c]	Mean of analytical SE	95% CI coverage [d]
$\theta = 0.2$ 50% valid IVs	50K	17 (4)	0.109	0.197	6.028	0.93
	100K	102 (8)	0.2	0.072	0.091	0.98
	200K	386 (12)	0.206	0.02	0.025	0.98
	500K	1151 (14)	0.209	0.01	0.011	0.93
	1000K	1802 (13)	0.206	0.007	0.008	0.94
$\theta = 0.2$ 25% valid IVs	50K	13 (3)	0.08	0.206	2.929	0.91
	100K	100 (7)	0.217	0.112	0.117	0.94
	200K	417 (13)	0.271	0.037	0.048	0.69
	500K	1156 (15)	0.217	0.016	0.02	0.92
	1000K	1826 (14)	0.211	0.009	0.012	0.94
$\theta = -0.2$ 50% valid IVs	50K	17 (4)	-0.035	0.2	8.648	0.9
	100K	102 (8)	-0.11	0.073	0.086	0.81
	200K	386 (12)	-0.162	0.019	0.024	0.71
	500K	1151 (14)	-0.18	0.01	0.011	0.6
	1000K	1802 (13)	-0.19	0.007	0.008	0.8
$\theta = -0.2$ 25% valid IVs	50K	13 (3)	-0.003	0.213	4.639	0.9
	100K	100 (7)	-0.053	0.117	0.272	0.82
	200K	417 (13)	-0.1	0.035	0.045	0.36
	500K	1156 (15)	-0.17	0.015	0.019	0.69
	1000K	1826 (14)	-0.185	0.01	0.011	0.86

[a] n_x: sample size of the study associated with X; sample size of the study associated with Y is $n_x/2$.
[b] IVs are defined as SNPs which reach genome-wide significance (z-test $p < 5\times10^{-8}$) in the study associated with X.
[c] Empirical SE of the causal estimate is calculated as the standard deviation of the causal estimates across 100 simulations.
[d] 95% confidence intervals are computed as $\hat{\theta}\pm1.96$ (standard error estimate).

Supplementary Table 2. Summary level data used in this paper.

Trait	Year published	Sample size	Ref	Accession date
**Exposure**				
BMI	2018	681,275	[1]	5/3/2018
Height	2018	693,529	[1]	6/5/2018
LDL	2013	188,577	[2]	5/3/2018
HDL	2013	188,577	[2]	5/3/2018
TG	2013	188,577	[2]	5/3/2018
Diastolic blood pressure	2017	317,756	[3]	5/19/2018
Systolic blood pressure	2017	317,754	[3]	5/19/2018
Age at menarche	2017	252,514	[4]	8/6/2018
Years of education	2018	766,345	[5]	9/4/2018
**Outcome**				
Coronary artery disease	2015	60,801/123,504[a]	[6]	5/4/2018
Breast cancer	2017	106,571/95,762[a]	[7]	5/28/2018
Major depressive disorder	2018	59,851/113,154[a]	[8]	5/5/2018

[a] cases/controls.

Supplementary Table 3. Estimates and 95% confidence intervals for causal effects across exposures. The effect estimates represent s.d. unit increase in the outcome per s.d. unit increase in the genetically determined level of the exposure.

Exposure	Outcome[a]	# of IVs[b]	MRMix	IVW	Weighted median	Weighted mode	Egger	LDSC[c]
BMI	LDL	964	0.06 [0.01, 0.11]	0.02 [-0.01, 0.06]	0.05 [0.01, 0.08]	0.07 [-0.07, 0.21]	-0.1 [-0.32, 0.12]	0.1
	HDL	964	-0.25 [-0.3, -0.2]	-0.23 [-0.26, -0.2]	-0.23 [-0.26, -0.19]	-0.13 [-0.3, 0.04]	-0.4 [-0.62, -0.18]	-0.22
	TG	964	0.23 [0.18, 0.28]	0.17 [0.13, 0.2]	0.21 [0.17, 0.25]	0.25 [0.08, 0.41]	0.13 [-0.1, 0.36]	0.04
	DBP	966	0.29 [0.24, 0.34]	0.26 [0.23, 0.29]	0.27 [0.25, 0.29]	0.22 [0.08, 0.36]	0.38 [0.21, 0.55]	0.32
	SBP	967	0.16 [0.08, 0.24]	0.15 [0.12, 0.17]	0.16 [0.13, 0.18]	0.19 [0.08, 0.3]	0.33 [0.16, 0.49]	0.2
	Age at menarche	969	-0.28 [-0.34, -0.22]	-0.32 [-0.35, -0.29]	-0.29 [-0.32, -0.26]	-0.22 [-0.31, -0.13]	-0.45 [-0.67, -0.22]	-0.35
LDL	DBP	153	0.02 [-0.02, 0.06]	0 [-0.03, 0.03]	-0.01 [-0.03, 0.02]	-0.02 [-0.08, 0.05]	0.04 [-0.1, 0.17]	-0.11
	SBP	153	0.04 [0, 0.08]	0.01 [-0.01, 0.04]	0.02 [-0.01, 0.04]	0.02 [-0.05, 0.09]	0.07 [-0.05, 0.19]	-0.08
HDL	DBP	197	0.02 [-0.01, 0.05]	-0.04 [-0.07, -0.01]	-0.02 [-0.04, 0.01]	0 [-0.04, 0.03]	0.05 [-0.08, 0.18]	-0.17
	SBP	199	-0.02 [-0.05, 0.01]	-0.07 [-0.1, -0.05]	-0.04 [-0.06, -0.01]	0 [-0.05, 0.05]	0.03 [-0.1, 0.16]	-0.13
TG	DBP	128	-0.01 [-0.06, 0.04]	0.01 [-0.03, 0.04]	-0.01 [-0.04, 0.02]	-0.04 [-0.09, 0.02]	0.06 [-0.1, 0.22]	0.07
	SBP	128	0.03 [-0.03, 0.09]	0.03 [0, 0.06]	0.02 [-0.01, 0.05]	0.02 [-0.05, 0.09]	0.07 [-0.08, 0.23]	0.06

[a] LDL: low-density lipoprotein cholesterol. HDL: high-density lipoprotein cholesterol. TG: triglycerides. DBP: diastolic blood pressure. SBP: systolic blood pressure.
[b] IVs are defined as SNPs which reach genome-wide significance (z-test $p < 5\times10^{-8}$) in the study associated with X.
[c] LDSC: LD score regression estimates of causal effects is defined as ρ_g/h_x^2, the ratio between the estimated genetic covariance and the estimated heritability of the exposure (see Supplementary Notes for details).

Supplementary Table 4. Reverse directional MR analysis for pairs of traits in Table 1 and Supplementary Table 3.

Exposure	Outcome[a]	# of IVs[b]	MRMix	IVW	Weighted median	Weighted mode	Egger	LDSC[c]
Coronary artery disease	BMI	41	0.04 [-0.01, 0.09]	-0.04 [-0.07, -0.01]	-0.03 [-0.05, -0.01]	-0.01 [-0.05, 0.03]	-0.05 [-0.27, 0.18]	0.2
	LDL	36	-0.04 [-0.1, 0.02]	-0.06 [-0.12, 0.01]	-0.07 [-0.12, -0.02]	-0.04 [-0.12, 0.03]	-0.22 [-0.7, 0.27]	0.09
	HDL	40	0.02 [-0.03, 0.07]	-0.07 [-0.13, -0.02]	-0.04 [-0.08, 0]	-0.02 [-0.1, 0.05]	0.05 [-0.35, 0.46]	-0.2
	TG	41	0.01 [-0.05, 0.07]	0.03 [-0.02, 0.08]	0.01 [-0.03, 0.05]	0 [-0.06, 0.07]	-0.31 [-0.64, 0.02]	0.17
	SBP	41	0 [-0.04, 0.04]	0.06 [0.01, 0.1]	0.02 [-0.01, 0.05]	0.01 [-0.04, 0.07]	-0.13 [-0.45, 0.18]	0.23
	DBP	41	-0.11 [-0.15, -0.07]	-0.02 [-0.08, 0.04]	-0.07 [-0.1, -0.03]	-0.09 [-0.13, -0.05]	-0.33 [-0.74, 0.09]	0.26
Breast cancer	BMI	83	0.02 [0, 0.04]	0 [-0.02, 0.02]	0 [-0.01, 0.02]	0.02 [-0.02, 0.05]	-0.03 [-0.19, 0.13]	-0.09
	Height	81	0.01 [-0.07, 0.09]	0 [-0.03, 0.04]	0.01 [-0.01, 0.03]	0.02 [-0.02, 0.05]	-0.04 [-0.3, 0.21]	0.07
	LDL	83	0 [-0.06, 0.06]	-0.01 [-0.04, 0.02]	-0.01 [-0.05, 0.02]	0 [-0.14, 0.14]	-0.02 [-0.28, 0.25]	0.01
	HDL	83	0.05 [-0.07, 0.17]	0.02 [-0.02, 0.05]	0.02 [-0.02, 0.06]	0.02 [-0.07, 0.11]	-0.09 [-0.35, 0.17]	0.07
	TG	83	-0.04 [-0.08, 0]	-0.02 [-0.05, 0.02]	-0.02 [-0.06, 0.02]	-0.04 [-0.12, 0.04]	0.13 [-0.16, 0.42]	-0.03
	Age at menarche	94	-0.01 [-0.05, 0.03]	0 [-0.03, 0.02]	-0.01 [-0.03, 0.01]	-0.03 [-0.08, 0.03]	-0.03 [-0.23, 0.18]	0.14
Major depressive disorder	BMI	3	-0.04 [-0.09, 0.01]	0.04 [0, 0.09]	0.01 [-0.06, 0.09]	-0.03 [-0.1, 0.04]	3.09 [0.52, 5.66]	0.06
	Years of education	4	-0.12 [-0.18, -0.06]	-0.02 [-0.23, 0.19]	-0.1 [-0.16, -0.05]	-0.12 [-0.16, -0.08]	-3.31 [-8.22, 1.59]	-0.14
LDL	BMI	149	-0.02 [-0.06, 0.02]	-0.04 [-0.06, -0.01]	-0.03 [-0.05, -0.02]	-0.03 [-0.07, 0.01]	-0.13 [-0.26, 0]	0.07
HDL	BMI	187	-0.04 [-0.07, -0.01]	-0.01 [-0.03, 0.02]	-0.02 [-0.04, 0]	-0.02 [-0.07, 0.02]	-0.02 [-0.13, 0.09]	-0.16
TG	BMI	126	-0.01 [-0.05, 0.03]	-0.02 [-0.05, 0.01]	-0.02 [-0.04, 0]	-0.02 [-0.05, 0.02]	0.06 [-0.1, 0.21]	0.03
DBP	BMI	220	-0.06 [-0.12, 0]	0.08 [0.04, 0.12]	0.03 [0, 0.05]	-0.02 [-0.09, 0.05]	-0.24 [-0.51, 0.03]	0.33
SBP	BMI	207	0.02 [-0.06, 0.1]	0.05 [0.01, 0.09]	0.02 [0, 0.05]	0.02 [-0.06, 0.11]	-0.3 [-0.56, -0.03]	0.21
Age at menarche	BMI	291	-0.09 [-0.13, -0.05]	-0.15 [-0.18, -0.12]	-0.12 [-0.14, -0.1]	-0.09 [-0.14, -0.05]	0.1 [-0.11, 0.3]	-0.26
DBP	LDL	222	-0.01 [-0.11, 0.09]	-0.07 [-0.12, -0.02]	-0.06 [-0.11, -0.01]	0.12 [-0.06, 0.3]	-0.09 [-0.41, 0.23]	-0.16
SBP	LDL	206	-0.02 [-0.07, 0.03]	-0.07 [-0.11, -0.02]	-0.06 [-0.11, -0.01]	-0.15 [-0.29, -0.02]	-0.54 [-0.86, -0.22]	-0.13
DBP	HDL	224	-0.06 [-0.14, 0.02]	-0.08 [-0.13, -0.03]	-0.07 [-0.12, -0.01]	0.05 [-0.1, 0.2]	0.27 [-0.09, 0.63]	-0.26
SBP	HDL	204	0.02 [-0.05, 0.09]	-0.06 [-0.12, -0.01]	-0.02 [-0.07, 0.04]	0 [-0.13, 0.13]	0.23 [-0.14, 0.61]	-0.2
DBP	TG	223	0.1 [-0.04, 0.24]	0.02 [-0.04, 0.07]	0.05 [-0.01, 0.11]	0.14 [-0.04, 0.32]	-0.37 [-0.74, 0.01]	0.1
SBP	TG	206	0.11 [0.01, 0.21]	0.04 [-0.02, 0.09]	0.07 [0.02, 0.13]	0.12 [-0.03, 0.27]	-0.09 [-0.43, 0.25]	0.09

[a] LDL: low-density lipoprotein cholesterol. HDL: high-density lipoprotein cholesterol. TG: triglycerides. DBP: diastolic blood pressure. SBP: systolic blood pressure [b]

[b] IVs are defined as SNPs which reach genome-wide significance (z-test $p < 5\times10^{-8}$) in the study associated with X.

[c] LDSC: LD score regression estimates of causal effects is defined as ρ_g/h_x^2, the ratio between the estimated genetic covariance and the estimated heritability of the exposure (see Supplementary Notes for details).

Supplementary Table 5. MRMix estimates under different SNP selection thresholds. Estimates of association coefficient for SNPs across two traits are simulated assuming an underlying four-component model for effect-size distribution (Scenario A, see **Methods**), where SNPs could have direct effects on neither traits, only on X, only on Y, or on both with the effects being correlated. The proportion of valid instruments, i.e. the SNPs which have only direct effects on X as a proportion of the total number of SNPs which are associated with X, is fixed at 50%. Mean (standard deviation) of causal estimates are reported over 100 simulations. The true causal effect is 0.2; the ratio of sample size of the study associated with X and Y (denoted by n_x and n_y respectively) is fixed at 2:1. Source data are provided as a Source Data file.

	n_x	**50K**	**100K**	**200K**	**500K**	**1000K**
SNP selection and effect estimation performed on the same dataset associated with X	MRMix p<0.005	0.05 (0.02)	0.1 (0.01)	0.13 (0.01)	0.17 (0.01)	0.18 (0.01)
	MRMix p<5e-04	0.11 (0.04)	0.16 (0.02)	0.19 (0.01)	0.19 (0.01)	0.2 (0.01)
	MRMix p<5e-06	0.16 (0.12)	0.19 (0.04)	0.2 (0.02)	0.2 (0.01)	0.2 (0.01)
	MRMix p<5e-08	0.14 (0.19)	0.19 (0.06)	0.2 (0.02)	0.2 (0.01)	0.2 (0.01)
	IVW	0.32 (0.09)	0.38 (0.04)	0.41 (0.02)	0.43 (0.02)	0.44 (0.01)
	Weighted median	0.31 (0.12)	0.32 (0.04)	0.32 (0.03)	0.3 (0.01)	0.29 (0.01)
	Weighted mode	0.24 (0.16)	0.21 (0.06)	0.2 (0.04)	0.2 (0.02)	0.2 (0.01)
	Egger	0.14 (1.66)	0.39 (0.31)	0.43 (0.11)	0.44 (0.06)	0.45 (0.04)
	Average # of IVs[b]	14 (4)	105 (10)	400 (18)	1134 (28)	1779 (32)
SNP selection and effect estimation performed on two independent datasets associated X[a]	MRMix p<0.005	0.15 (0.06)	0.19 (0.03)	0.2 (0.02)	0.2 (0.01)	0.2 (0.01)
	MRMix p<5e-04	0.21 (0.09)	0.22 (0.03)	0.21 (0.02)	0.2 (0.01)	0.2 (0.01)
	MRMix p<5e-06	0.18 (0.18)	0.23 (0.04)	0.22 (0.02)	0.21 (0.01)	0.2 (0.01)
	MRMix p<5e-08	0.11 (0.25)	0.22 (0.06)	0.22 (0.02)	0.21 (0.01)	0.2 (0.01)
	IVW	0.44 (0.13)	0.44 (0.05)	0.44 (0.03)	0.44 (0.02)	0.44 (0.01)
	Weighted median	0.4 (0.16)	0.37 (0.06)	0.35 (0.03)	0.31 (0.02)	0.29 (0.01)
	Weighted mode	0.32 (0.19)	0.25 (0.07)	0.21 (0.04)	0.2 (0.02)	0.2 (0.01)
	Egger	0.27 (0.42)	0.25 (0.17)	0.32 (0.09)	0.39 (0.05)	0.42 (0.04)
	Average # of IVs	14 (4)	104 (10)	400 (17)	1138 (28)	1777 (33)

[a] The dataset for SNP selection and the dataset for effect estimation have the same sample size.
[b] IVs are defined as SNPs which reach genome-wide significance (z-test $p < 5\times10^{-8}$) in the study associated with X.

Supplementary References

1. Yengo, L. et al. Meta-analysis of genome-wide association studies for height and body mass index in ~700000 individuals of European ancestry. *Hum Mol Genet* **27**, 3641-3649 (2018).
2. Willer, C. J. et al. Discovery and refinement of loci associated with lipid levels. *Nat Genet* **45**, 1274 (2013).
3. Neale, B. Rapid GWAS of thousands of phenotypes for 337,000 samples in the UK Biobank. http://www.nealelab.is/blog/2017/7/19/rapid-gwas-of-thousands-of-phenotypes-for-337000-samples-in-the-uk-biobank. (2017).
4. Day, F. R. et al. Genomic analyses identify hundreds of variants associated with age at menarche and support a role for puberty timing in cancer risk. *Nat Genet* **49**, 834-834 (2017).
5. Lee, J. J. et al. Gene discovery and polygenic prediction from a genome-wide association study of educational attainment in 1.1 million individuals. *Nat Genet* **50**, 1112 (2018).
6. The CARDIoGRAMplusC4D Consortium. A comprehensive 1000 Genomes–based genome-wide association meta-analysis of coronary artery disease. *Nat Genet* **47**, 1121-1121 (2015).
7. Michailidou, K. et al. Association analysis identifies 65 new breast cancer risk loci. *Nature* **551**, 92-92 (2017).
8. Wray, N. R. et al. Genome-wide association analyses identify 44 risk variants and refine the genetic architecture of major depression. *Nat Genet* **50**, 668 (2018).
