# Supplementary material for: Mendelian randomization analysis using mixture models for robust and efficient estimation of causal effects
Source: Nat Commun. 2019 Apr 26;10:1941. doi: 10.1038/s41467-019-09432-2 (PMC6486646; doi:10.1038/s41467-019-09432-2)
Supplement: Supplementary file 3 — Reporting Summary [file 41467_2019_9432_MOESM3_ESM.pdf]

nature research

Corresponding author(s): Nilanjan Chatterjee

Reporting Summary

Nature Research wishes to improve the reproducibility of the work that we publish. This form provides structure for consistency and transparency in reporting. For further information on Nature Research policies, see Authors & Referees and the Editorial Policy Checklist.

Statistical parameters

When statistical analyses are reported, confirm that the following items are present in the relevant location (e.g. figure legend, table legend, main text, or Methods section).

n/a	Confirmed	
☐	☒	The exact sample size (n) for each experimental group/condition, given as a discrete number and unit of measurement
☒	☐	An indication of whether measurements were taken from distinct samples or whether the same sample was measured repeatedly
☒	☐	The statistical test(s) used AND whether they are one- or two-sided<br>*Only common tests should be described solely by name; describe more complex techniques in the Methods section.*
☒	☐	A description of all covariates tested
☐	☒	A description of any assumptions or corrections, such as tests of normality and adjustment for multiple comparisons
☐	☒	A full description of the statistics including central tendency (e.g. means) or other basic estimates (e.g. regression coefficient) AND variation (e.g. standard deviation) or associated estimates of uncertainty (e.g. confidence intervals)
☒	☐	For null hypothesis testing, the test statistic (e.g. F, t, r) with confidence intervals, effect sizes, degrees of freedom and P value noted<br>*Give P values as exact values whenever suitable.*
☒	☐	For Bayesian analysis, information on the choice of priors and Markov chain Monte Carlo settings
☒	☐	For hierarchical and complex designs, identification of the appropriate level for tests and full reporting of outcomes
☐	☒	Estimates of effect sizes (e.g. Cohen's d, Pearson's r), indicating how they were calculated
☐	☒	Clearly defined error bars<br>*State explicitly what error bars represent (e.g. SD, SE, CI)*

Our web collection on statistics for biologists may be useful.

Software and code

Policy information about availability of computer code

Data collection	No software code was used.
Data analysis	Software code available at: https://github.com/gqi/MRMix

For manuscripts utilizing custom algorithms or software that are central to the research but not yet described in published literature, software must be made available to editors/reviewers upon request. We strongly encourage code deposition in a community repository (e.g. GitHub). See the Nature Research guidelines for submitting code & software for further information.

Data

Policy information about availability of data

All manuscripts must include a data availability statement. This statement should provide the following information, where applicable:

- Accession codes, unique identifiers, or web links for publicly available datasets
- A list of figures that have associated raw data
- A description of any restrictions on data availability

The data used in this study are publicly available at the URLs below.
1000 Genomes Phase 3 European sample, HapMap3 SNP list, https://data.broadinstitute.org/alkesgroup/LDSCORE/;
GIANT Consortium (BMI and height) summary statistics, http://portals.broadinstitute.org/collaboration/giant/index.php/GIANT_consortium_data_files;

Global Lipids Genetics Consortium (cholesterol traits), http://csg.sph.umich.edu/abecasis/public/lipids2013/;
Neal lab UK Biobank GWAS (blood pressure summary statistics), http://www.nealelab.is/blog/2017/7/19/rapid-gwas-of-thousands-of-phenotypes-for-337000-samples-in-the-uk-biobank;
ReproGen Consortium (age at menarche), http://www.reprogen.org/data_download.html;
Social Science Genetic Association Consortium (SSGAC), https://www.thessgac.org/data;
CARDIoGRAMplusC4D Consortium (coronary artery disease), http://www.cardiogramplusc4d.org/data-downloads/;
Breast Cancer Association Consortium (BCAC) summary statistics, http://bcac.ccge.medschl.cam.ac.uk/bcacdata/oncoarray/gwas-icogs-and-oncoarray-summary-results/;
Psychiatric Genomics Consortium (PGC), https://www.med.unc.edu/pgc/results-and-downloads/downloads.
The source data underlying Figures 1b, 2, 3, Supplementary Figures 1-9 and Supplementary Tables 1 and 5 are provided as a Source Data file. All other relevant data are available upon request.

Field-specific reporting

Please select the best fit for your research. If you are not sure, read the appropriate sections before making your selection.

☒ Life sciences ☐ Behavioural & social sciences ☐ Ecological, evolutionary & environmental sciences

For a reference copy of the document with all sections, see nature.com/authors/policies/ReportingSummary-flat.pdf

Life sciences study design

All studies must disclose on these points even when the disclosure is negative.

Sample size	We used pubicly available data that had pre-determined sample sizes.
Data exclusions	We used summary-level data and thus has no ability to remove subjects from analysis. We filtered SNPs following guidelines pre-established by LD-score regression. The filtering steps are described in Methods.
Replication	The final software code for analysis is made publicly available through GitHub repository. This code can be used to analyze the publicly available datasets to reproduce our data analysis results.
Randomization	NA
Blinding	NA

Reporting for specific materials, systems and methods

Materials & experimental systems

n/a	Involved in the study	
☒	☐	Unique biological materials
☒	☐	Antibodies
☒	☐	Eukaryotic cell lines
☒	☐	Palaeontology
☒	☐	Animals and other organisms
☒	☐	Human research participants

Methods

n/a	Involved in the study	
☒	☐	ChIP-seq
☒	☐	Flow cytometry
☒	☐	MRI-based neuroimaging
